# Advances in Multi-Omics Approaches for Molecular Breeding of Black Rot Resistance in *Brassica oleracea* L.

**DOI:** 10.3389/fpls.2021.742553

**Published:** 2021-12-06

**Authors:** Ranjan K. Shaw, Yusen Shen, Jiansheng Wang, Xiaoguang Sheng, Zhenqing Zhao, Huifang Yu, Honghui Gu

**Affiliations:** Institute of Vegetables, Zhejiang Academy of Agricultural Sciences, Hangzhou, China

**Keywords:** *Brassica oleracea*, black rot, *Xanthomonas campestris* pv. *campestris*, omics, genomics, transcriptomics, proteomics, metabolomics

## Abstract

*Brassica oleracea* is one of the most important species of the *Brassicaceae* family encompassing several economically important vegetables produced and consumed worldwide. But its sustainability is challenged by a range of pathogens, among which black rot, caused by *Xanthomonas campestris* pv. *campestris* (*Xcc*), is the most serious and destructive seed borne bacterial disease, causing huge yield losses. Host-plant resistance could act as the most effective and efficient solution to curb black rot disease for sustainable production of *B. oleracea*. Recently, ‘omics’ technologies have emerged as promising tools to understand the host-pathogen interactions, thereby gaining a deeper insight into the resistance mechanisms. In this review, we have summarized the recent achievements made in the emerging omics technologies to tackle the black rot challenge in *B. oleracea*. With an integrated approach of the omics technologies such as genomics, proteomics, transcriptomics, and metabolomics, it would allow better understanding of the complex molecular mechanisms underlying black rot resistance. Due to the availability of sequencing data, genomics and transcriptomics have progressed as expected for black rot resistance, however, other omics approaches like proteomics and metabolomics are lagging behind, necessitating a holistic and targeted approach to address the complex questions of *Xcc-Brassica* interactions. Genomic studies revealed that the black rot resistance is a complex trait and is mostly controlled by quantitative trait locus (QTL) with minor effects. Transcriptomic analysis divulged the genes related to photosynthesis, glucosinolate biosynthesis and catabolism, phenylpropanoid biosynthesis pathway, ROS scavenging, calcium signalling, hormonal synthesis and signalling pathway are being differentially expressed upon *Xcc* infection. Comparative proteomic analysis in relation to susceptible and/or resistance interactions with *Xcc* identified the involvement of proteins related to photosynthesis, protein biosynthesis, processing and degradation, energy metabolism, innate immunity, redox homeostasis, and defence response and signalling pathways in *Xcc*–*Brassica* interaction. Specifically, most of the studies focused on the regulation of the photosynthesis-related proteins as a resistance response in both early and later stages of infection. Metabolomic studies suggested that glucosinolates (GSLs), especially aliphatic and indolic GSLs, its subsequent hydrolysis products, and defensive metabolites synthesized by jasmonic acid (JA)-mediated phenylpropanoid biosynthesis pathway are involved in disease resistance mechanisms against *Xcc* in *Brassica* species. Multi-omics analysis showed that JA signalling pathway is regulating resistance against hemibiotrophic pathogen like *Xcc*. So, the bonhomie between omics technologies and plant breeding is going to trigger major breakthroughs in the field of crop improvement by developing superior cultivars with broad-spectrum resistance. If multi-omics tools are implemented at the right scale, we may be able to achieve the maximum benefits from the minimum. In this review, we have also discussed the challenges, future prospects, and the way forward in the application of omics technologies to accelerate the breeding of *B. oleracea* for disease resistance. A deeper insight about the current knowledge on omics can offer promising results in the breeding of high-quality disease-resistant crops.

## Introduction

*Brassica oleracea* is one of the most important species of the *Brassicaceae* family, encompassing several economically important vegetables such as cabbage, cauliflower, broccoli, kale, kohlrabi, and brussels sprouts. Among all these vegetables, cabbage, and cauliflower are widely produced while broccoli is relatively new and is emerging as a most sought vegetable in several countries. Brussels sprouts, kale, and kohlrabi though are not popular like the other three vegetables but are important on a regional or country basis (Quiros and Farnham, 2011). China and India are the highest producer of cauliflowers, broccoli, cabbages, and other *Brassica* vegetables with a total production of 44.85 and 18.21 million tonnes, respectively (FAOSTAT, 2019), followed by several other countries of Asia and Europe. Human selection has been helpful in creating a wide morphological variation within *B. oleracea species. B. oleracea* vegetables are extremely healthy and rich in nutrients with optimal health benefits. All these vegetables contain variable amount of vitamin, fiber, minerals, and useful phytochemicals (Cartea et al., 2011a; Gupta, 2011). In addition, this vegetable group is a rich source of sulfur-containing secondary metabolites, called glucosinolates (Kapusta-Duch et al., 2012) which possess anti-cancer properties. *B. oleracea* crops are highly sensitive to biotic stresses (fungal, bacterial, and viral) resulting in severe yield and quality losses. Among all, black rot is the most serious, destructive bacterial disease prevalent in many countries where *B. oleracea* crops are widely grown (Williams, 1980; Singh et al., 2011). Black rot was first reported in cabbage (Garman, 1894) and has spread to all regions of the world. So, black rot has a wide geographical distribution across the continents including Asia (China, India, Nepal, Taiwan), Europe (Italy, Spain, France, Belgium, Germany, Sweden, Hungary, Netherlands, Portugal, United Kingdom), Africa (Ethiopia, South Africa), North America (United States, Canada), South America (Brazil), and Australia (Saharan, 1993; Quiros and Farnham, 2011; Mulema et al., 2012; Singh et al., 2016; Akhtar et al., 2017) causing huge yield losses in cruciferous vegetables. The disease is also harmful in a way that it makes the plants prone to *Alternaria blight* attack (Sharma et al., 1991). The disease causes considerable yield losses up to 50–60% in cauliflower and affects the quality of the curd (Williams, 1980; Kashyap and Dhiman, 2010; Dhar and Singh, 2014), reducing its marketability. Several management strategies, including good cultural practices such as crop rotation, crop residue management, avoidance of water lodging, hot water and bactericide (e.g., sodium hypochlorite, hydrogen peroxide) treatment of seeds, and planting of disease-free materials (seeds or transplants), use of resistant varieties could be followed to reduce the spread of the disease. Among all, growing potentially *Xcc*-resistant cultivars could be the sustainable approach within the integrated management of disease and host–plant resistance can act as a key strategy to curb black rot disease. Advances in molecular biology and sequencing technologies in the post-genomics era can be exploited as powerful tools to tackle this challenge.

The recent development of genomic resources has led to the development of genetic/physical maps leading to the identification of several quantitative trait loci (QTLs) and candidate genes responsible for black rot resistance in *B. oleracea*. Rapid progresses were made in the ‘omics’ technologies at the genomic, transcriptomic, proteomic, and metabolomic levels permitting the researchers to identify the genetic underpinnings, i.e., genes to improve the productivity and quality of the crops. The emergence of omics technologies has enabled the researchers to have a direct and unbiased monitoring of the factors affecting the crop growth, yield, metabolism, biotic, and abiotic stresses (Setia and Setia, 2008). It has helped in the investigation of the biology behind several agronomic traits at the physiological, biochemical, and molecular levels accelerating the crop production. Omics approaches have shaped our understanding on the complex interactions between genes, proteins, and metabolites within the resulting phenotype (Emon, 2016). Omics helps in understanding the linkage between the genotypes and phenotypes and in studying the entire pathway eliciting the phenotypes (Guillemin et al., 2016). The knowledge generated from omics could be useful in understanding the complex pathways involved in disease resistance. Technological advances have driven the omics technologies to be cost-effective and carryout high-throughput analysis of biological samples (Hasin et al., 2017).

**FIGURE 1:**
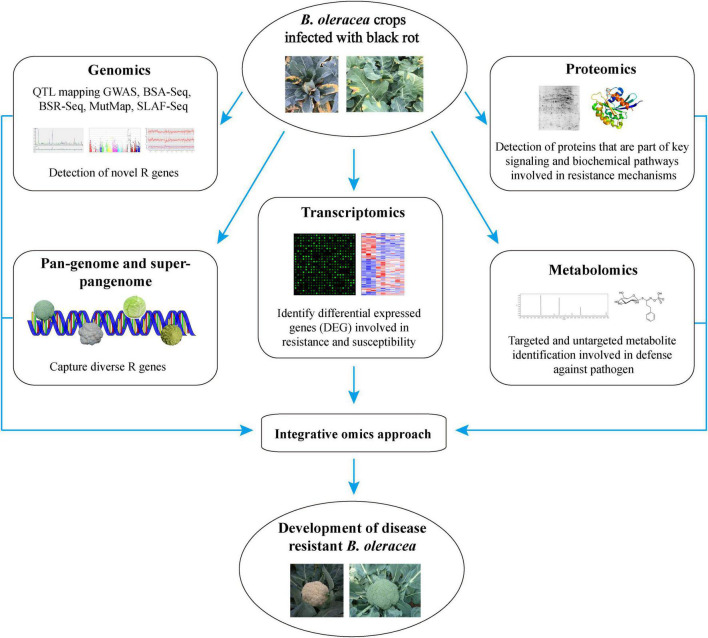
The application of ‘multi-omics’ technologies to develop *Xcc* resistant *B. oleracea* and to understand the mechanisms of disease resistance.

The recent advancement in next-generation sequencing (NGS) technologies has led to the publishing of many research articles in the field of different omics techniques. Consequently, a huge number of multi-omics data has been generated at the DNA, RNA, protein, and metabolite levels (Choi, 2019) which could be analysed to decipher the complex plant defence systems. In this era of big biological data, omics technologies are widely used for crop improvements in several major agricultural crops such as wheat (Alotaibi et al., 2021), allium (Khandagale et al., 2020), rice (Peng et al., 2020), sesamum (Dossa et al., 2017), and have revolutionized the modern agricultural research. This, in turn, are creating unprecedented opportunities for the plant researchers who can use the multi-omics data to decipher the multigenicity of biotic and abiotic plant stress responses, protein and metabolite profiles, and their dynamic changes in plants. The recent achievements in ‘omics’ technologies have opened up a plethora of possibilities to understand the complex *B. oleracea-Xcc* interaction to develop resistant *B. oleracea* crops (Figure 1). However, many pitfalls and limitations exist to integrate and use these approaches, which need to be taken care of. Here, in this review, we are going to summarize the recent achievements made in the molecular breeding and emerging omics technologies to tackle the black rot challenge in *B. oleracea*. We have also discussed the challenges, future prospects, and the way forward in the application of omics technologies for accelerating the breeding of *B. oleracea* crops for disease resistance.

## Black Rot

Black rot is one of the most serious and destructive bacterial diseases of *B. oleracea* prevalent in all agro-climatic zones of the world (Williams, 1980; Stall et al., 1993; Taylor et al., 2002; Singh and Dhar, 2011). Black rot caused by *Xanthomonas campestris* pv. *campestris* (Pammel) Dowson (hereinafter it will be referred as *Xcc*) is a seed borne, gram-negative, aerobic, and vascular bacterium. Under favourable conditions of plenty rainfall, high humidity, and average temperature between 25 and 30°C, the disease becomes more harmful leading to higher yield losses. Due to the curd infection, seed yield is also reduced drastically in cauliflower (Patel et al., 1970). *Xcc* attack reportedly decreases the biomass of *B. oleracea* seedlings at least 28 days after infection (Vega-Álvarez et al., 2021).

### Infection Process, Symptoms, and Disease Cycle of *Xcc* in *Brassica*

**FIGURE 2:**
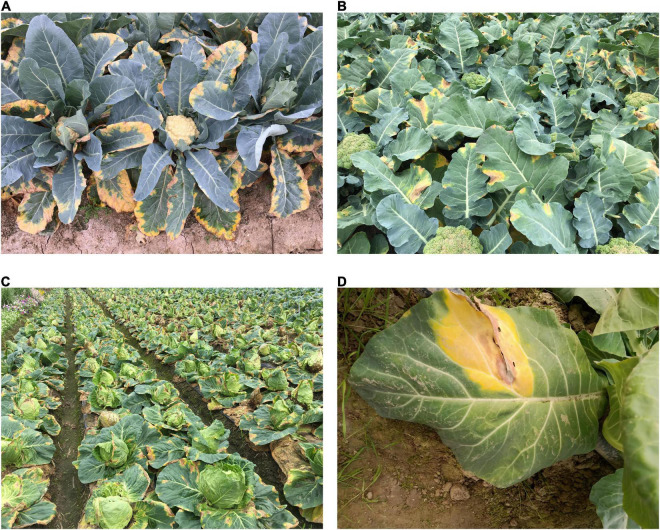
Different *Brassica oleracea* varieties infested with *Xanthomonas campestris* pv. *campestris* [**(A)** Cauliflower plants with severe *Xcc* infection, **(B)** Broccoli plants with typical symptoms of *Xcc*, **(C)** Cabbage plants with heavy *Xcc* infestation **(D)** Cauliflower leaves with characteristic V-shaped lesion caused by *Xcc* infection].

*Xcc* infection can occur at any developmental stages of the plant, starting with germination of the infected seeds to maturity. *Xcc* primarily spreads from the infected seeds, which is a major route of disease transmission. However, black rot can also be transmitted through infested soil, crop residues, and by various environmental and mechanical means via wind, insects, aerosols, irrigation water, rain, and farm equipments (Vicente and Holub, 2013). The pathogen can survive longer in plant debris in soil than as free-living cells for up to 2 years. The germination of colonised seeds leads to the infection of the seedlings. The bacteria mostly enter the plants through the hydathodes on the leaf margins although it can also invade the plant through the wounds caused by machinery, insects, animals, rain, irrigation, and wind, etc. Through these entry points, the bacteria spread intercellularly, colonize the mesophyll first, and then gain access to the plant vascular systems and multiply in the vessels leading to the rapid spreading of systemic host infection. At the same time, the xylem disintegrates, spreading the bacteria between the surrounding parenchyma cells, killing the cells, and causing cavities to be formed (Agrios, 2005). The symptoms are manifested by very distinctive appearance, i.e., V-shaped chlorotic to necrotic yellow lesions originating from the leaf margins and progressing toward the middle vein of the leaves. Also, the symptoms include the darkening of veins of the leaves and of the vascular tissue of the stem. Due to necrosis, the leaves fall prematurely, and systemic infection may lead to stunted growth of the plants. During warm and humid climates, *Xcc* thrives as a severe disease agent and the bacteria often ooze out to the surface of the leaves through the hydathodes/wounds and subsequently spread in droplets of guttation to the neighbouring plants by wind, rain, or water splashes. Black rot affecting different varieties of *B. oleracea* with the typical V-shaped lesion is depicted in Figures 2A–D.

Several pathogenic races of *Xcc* were reported in different *Brassica* species and Kamoun et al. (1992) first proposed the race structure of *Xcc*. Initially, six races (1–6) were identified by Vicente et al. (2001). Later, three more races (7–9) were identified by Fargier and Manceau (2007) and recently, two novel races, race 10 and 11 were reported in Portugal (Cruz et al., 2017). Presently, eleven different physiological races infecting *Brassica* species have been reported (Vicente et al., 2001; Fargier and Manceau, 2007; Cruz et al., 2017), indicating the complexity of *Xcc*. Among all, races 1 and 4 are the most aggressive and predominant worldwide (Lema et al., 2012a; Vicente and Holub, 2013) though their frequencies in *B. oleracea* varies with the geographical region.

## Resistance Sources and Genetics of Resistance

As breeding of resistant varieties is one of the effective measures to control black rot, several studies were conducted to identify diverse resistance sources in *B. oleracea*. Since the first reporting of ‘Early Fuji’ as resistant to black rot (Bain, 1952), numerous resistance sources have been identified in *B. oleracea*. During the beginning of the 21st century, *Xcc* was differentiated in to pathogenic races and the researchers have been screening and identifying resistance sources specific to different races of *Xcc*. But again, the problem is that a single resistant cultivar/line may not provide resistance to all the prevalent races of *Xcc*. As races 1 and 4 are the most aggressive and prevalent in *B. oleracea*, extensive screening of the accessions was carried out to find out the novel resistance sources. Taylor et al. (2002) screened a large set of accessions of *B. oleracea* against race 1 and only a single accession showed partial resistance, indicating the rare existence of resistance sources for race 1. Still, efforts were made to identify the resistance to race 1 and very recently, Kong et al. (2021) evaluated a worldwide collection of 162 cabbage accessions against race 1 and only four germplasms, including two inbred lines (‘05-574-323’ and ‘MD219’) and two hybrids (‘Qinglian’ and ‘Dadilv 2’), were found to be highly resistant. In another study, out of 27 cabbage inbred lines, only one line (SCNU-C-4074) showed resistance to *Xcc* race 1 (Afrin et al., 2018a). Griesbach et al. (2003) identified one highly resistant cabbage, ‘AU4518,’ against race 1. In addition, different authors have screened and identified few accessions showing resistance to race 1 (Jensen et al., 2005; Lema et al., 2012b; Saha et al., 2016).

On contrary, screening of *B. oleracea* against *Xcc*4 identified only a few resistance sources. Taylor et al. (2002) couldn’t identify a single accession conferring resistance to race 4 while screening a large set of *B. oleracea* accessions against a range of races of black rot. Recently, 26 cauliflower and six related wild species were screened against *Xcc*4 to identify novel sources of resistance. Among them, only one cauliflower inbred line (Boc4601) and three wild accessions (PI435896, UNICT5168, UNICT5169) showed better resistance (Sheng et al., 2020). Several scholars have screened and identified few resistant lines against *Xcc*4 in *B*. *oleracea* (Griesbach et al., 2003; Lema et al., 2012b; Saha et al., 2016). In contrast, comparatively a greater number of accessions of *B*. *oleracea* have been identified showing resistance to other races such as 1, 2, 3, 5, 6, and 7 (Afrin et al., 2018a), and races 2, 3, 5, and 6 (Taylor et al., 2002). Resistance to races 3 and 5 is common in *B. oleracea*, especially in cauliflower (Taylor et al., 2002).

The rare existence of resistance to races 1 and 4 in the ‘C’ genome of *B. oleracea* (Taylor et al., 2002; Vicente and Holub, 2013) complicates the efficient control of black rot, necessitating to explore the novel sources of resistance in the related *Brassica* species. Most common and potentially useful sources of black rot resistance is available in the ‘A’ and ‘B’ genome of *Brassica* species. Several authors have reported the race-specific resistance to both races 1 and 4 of in related *Brassica* species such as *B*. *nigra*, *B*. *rapa*, *B*. *carinata*, and *B*. *juncea* (Ignatov et al., 2000; Taylor et al., 2002; Vicente et al., 2002; Tonguç and Griffiths, 2004; Griffiths et al., 2009). According to Taylor et al. (2002), resistance to races 1 and 4 were present in a high proportion in the ‘B’ genomes (*B. nigra*, *B. carinata*, *B. juncea*) while strong resistance to race 4 has an ‘A’ genome origin (*B. rapa*, *B. napus*). The wild relatives of *Brassica* crops also could provide useful and durable sources of black rot resistance.

It is noteworthy that there could be certain limitations in using race-specific resistance materials in resistance breeding if only one specific resistance is prevalent in a target growing area. So, for durable resistance, accessions with broad-spectrum and race non-specific resistance are desirable (Taylor et al., 2002). In cauliflower, several race non-specific resistance sources have been identified (Sharma et al., 1972, 1977, 1995, 2003; Pandey et al., 2003; da Silva et al., 2015; Chatterjee et al., 2018). Also, in the ‘A’ and ‘B’ genomes of *Brassica* species, several durable resistance sources against *Xcc* have been reported (Westman et al., 1999; Taylor et al., 2002; Vicente and Holub, 2013; Dey et al., 2015; Lema et al., 2015; Sharma et al., 2016). Although the non-specific resistance is quantitative and durable, this is more difficult to manage and transfer between the cultivars.

Knowledge on the genetics of resistance guides in the resistance breeding to combat plant diseases. In *B*. *oleracea*, contradictory reports of the inheritance pattern of resistance genes in different genetic backgrounds and for different races have made the breeding for black rot resistance a challenging task. Worldwide, the widespread and predominant races 1 and 4 in *B*. *oleracea* collectively constitutes almost 94% of black rot disease (Vicente et al., 2001). Inheritance studies in *B*. *oleracea* varieties indicated the resistance to race 1 is controlled by a single dominant gene (Ignatov et al., 1998; Saha et al., 2014a,b, 2016), quantitative and recessive gene (Vicente et al., 2002), polygenes (Tonu et al., 2013), and a pair of additive major genes and additive-dominant multiple gene (Kong et al., 2021).

Resistance to race 4 was found to be governed by a single dominant gene (Vicente et al., 2002; Tonguç et al., 2003). But a recent study showed the prevalence of quantitative resistance against *Xcc*4 in an F_2_ population developed by a cross between wild species (*Brassica montana*) and cauliflower breeding line (Sheng et al., 2020). Resistance to race 3 of *Xcc* was controlled by a single dominant locus (*Xca3*) in doubled haploid line BOH 85c and PI 436606 of *B*. *oleracea*, whereas in Badger Inbred-16, the resistance to race 3 was found to be quantitative and recessive indicating the effect of genetic background (Vicente et al., 2002). Also, the same genotype showed different modes of inheritance to different races of black rot. Resistance of cabbage genotype ‘PI436606’ to *Xcc* race 1 and 3 was controlled by a single dominant gene (Ignatov et al., 1998; Vicente et al., 2002) whereas resistance to race 5 was reported to be controlled by a single recessive gene (Ignatov et al., 1998). Apart from this, the inheritance studies in cauliflower and cabbage have reported different modes of inheritance against black rot (without race information), such as single dominant gene (Jamwal and Sharma, 1986; Kaur et al., 2009), single recessive gene (Dickson and Hunter, 1987), polygenic dominant gene (Sharma et al., 1972; Tewari et al., 1979; Thakur et al., 2003), major genes with recessive and dominant modifiers (Williams et al., 1972), and non-additive genes (Pandey et al., 1995). Taken together, the genetics of black rot resistance is complex in *B*. *oleracea* and is genetically diverse. The inheritance pattern shows that both qualitative with a race-specific manner and quantitative resistance genes are responsible for black rot resistance.

## Advances in Omics Technologies

### Genomics

Genomics pertains to the study of all the genes in a genome, including the identification of gene sequences, gene structures, and annotations. It plays an important role in discovering the genetic variation underlying important traits and contribute to the genetic improvement of crop species. Rapid progress in the NGS technologies has expanded our ability to understand the whole genome and helps in bridging the gap between the genotype and phenotype. Genomic revolution has led to the generation of whole-genome sequences, expressed sequence tags (ESTs), large-insert genomic libraries, high density genetic maps, and millions of molecular markers which could be used for bi-parental/association mapping, cloning of genes/QTLs, and genomic selection, etc. for agronomically important traits in different crops. The identification of QTLs, allelic variation in the genes governing the trait of interest will enhance the possibilities of improvement of *Brassica* species especially for disease resistance.

#### Genome Assembly and Pangenomics

Advancement in the NGS technologies has led to the sequencing of crop genomes of several *Brassica* species, such as *B. oleracea* (Liu et al., 2014; Parkin et al., 2014; Belser et al., 2018; Sun et al., 2019), *B. rapa* (Wang et al., 2011), *B. nigra* (Yang et al., 2016), *B. napus* (Chalhoub et al., 2014), and *B. juncea* (Yang et al., 2016). Several long-read sequencing technologies such as PacBio Single Molecule Real-Time sequencing (SMRT) (Roberts et al., 2013) and Oxford Nanopore Technologies (Jain et al., 2016) have revolutionized the *Brassica* genomics. The *Brassica* database, BRAD^1^, provides the information on the genome assemblies, predicted gene models, and gene annotations of 25 *Brassica* species (Cheng et al., 2011), helping plant scientists and breeders to efficiently use the information to understand the complex mechanisms underlying disease resistance. The molecular aspects of *B. oleracea*–pathogen interactions could be revealed by using high-quality reference genome assemblies generated for different morphotypes of *B. oleracea* over the past several years including kale (Parkin et al., 2014), cabbage (Cai et al., 2020; Lv et al., 2020; Guo et al., 2021), cauliflower (Sun et al., 2019; Guo et al., 2021), and broccoli (Belser et al., 2018). But the high-quality genome assemblies of the reference genome of *B. oleracea* may not represent all the morphotypes and capture only a fraction of them such as inflorescence in ‘C-8’ (Sun et al., 2019) and leafy type in ‘TO1000’ (Parkin et al., 2014) leaving other morphotypes, such as lateral leaf buds (brussels sprouts) and tuberous stems (kohlrabi) not having genome assemblies. This resulted in missing out of genetic diversity in *B. oleracea* species which could have been the potential source of genomic variation associated with black rot resistance.

Pangenome analysis in *B. oleracea* allows the identification of genes from a gene pool represented by many lines of the given species (Tao et al., 2019; Bayer et al., 2020) and may lead to the identification of orthologous genes in *Brassica* species (Golicz et al., 2016). The idea of pangenomics could help in overcoming the limitation of dependence on a single reference genome. The pangenome analysis of *B. oleracea* varieties revealed that in many genomes, a large proportion of the disease resistance genes were not present in all the lines (Golicz et al., 2016) suggesting the variable nature of R-genes. This may result in the loss of many candidate R-genes from a single reference genome. Interestingly, the pangenome study of *B. oleracea* found the wild relative (*B. macrocarpa*) harbouring the most resistance gene analog (RGA) indicating that the genetic resources of wild species of *Brassica* could be the repository of novel R-genes (Golicz et al., 2016; Bayer et al., 2019). Through pangenomic approach, Bayer et al. (2019) identified 37 RGA candidates within QTL regions associated with black rot and sclerotinia resistance in *B. oleracea*. The identified RGA candidates were not present in a single reference assembly indicating the requirement of a pangenome to identify the candidate genes for breeding of improved cultivars. The authors also revealed that RGA candidates differed between lines in *B. oleracea* and the single-nucleotide polymorphisms (SNPs) and presence/absence variants (PAV) drove RGA diversity using separate mechanisms.

Recently, Khan et al. (2020) reported a super-pangenome which included the genomes of wild relatives and different species within a genus which could be replicated in *Brassica* species. This may allow the broadening of the *Brassica* gene pool and will help in the identification of novel candidate resistance genes for several diseases including black rot by capturing the maximum genomic variation present within the *Brassica* species.

#### Identification of Quantitative Trait Loci for Black Rot Resistance

To exploit the genomic technologies in breeding programme, mapping of QTLs governing the desired traits and information about the allelic variation of genes underlying the target traits is crucial. Several advanced molecular breeding techniques such as marker-assisted selection (MAS), marker-assisted backcrossing (MABC), marker-assisted recurrent selection (MARS), and marker-assisted gene pyramiding (Collard and Mackill, 2008; Ye and Smith, 2008; Ribaut et al., 2010; Ragimekula et al., 2013) could help in achieving durable resistance against black rot using the latest genomic technologies. Progress in the NGS technologies have fast-tracked the identification of markers co-segregating with genes of interest. Identification of QTLs help in exploiting the closely linked markers through marker-assisted selection in breeding programme (Collard et al., 2005; Collard and Mackill, 2008) and permits the validation of QTLs and its effect across a range of environments and genetic backgrounds.

As discussed earlier, races 1 and 4 of black rot is considered as the most virulent and widespread races in *B*. *oleracea* (Lema et al., 2012a; Vicente and Holub, 2013). So, several studies were framed to identify the R-genes/QTLs and markers linked to *Xcc*1 and *Xcc*4 resistance in *B. oleracea*, most importantly in cauliflower by different research groups (Table 1). Several random amplified polymorphic DNA (RAPD) markers linked to *Xcc*1 resistance locus were reported by various researchers. Saha et al. (2014b) mapped a *Xcc*1 resistance locus, *Xca1bo* on chromosome 3 in Indian cauliflower by bulk segregant analysis. Two markers (RAPD 04_833_ and ISSR 11_635_) were found flanking the resistance locus at 1.6-cM interval. Based on sequence homology with *B. rapa* genome, the location of *Xca1bo* was deduced to chromosome 3 in *B. oleracea*. The identified markers have the potential to be used in marker-assisted backcross breeding programme for introgression of the black rot (race 1) resistance. Again, Saha et al. (2014a) identified three RAPD markers (OPO-048_33_, OPAW-202_538_, and OPG-25_625_) controlling resistance to *Xcc* race 1 which were associated in coupling phase to the resistance allele and was found co-segregating with the black rot resistance gene. RAPD markers are dominant in nature, so they need to be converted into sequence characterized amplified region (SCAR) markers for utility purpose. Two sequence characterized amplified regions (SCAR) markers (ScOPO-04833 and ScPKPS-11635) were identified in close linkage with the black rot resistance locus, *Xca1Bo* (resistance to *Xcc*1), in cauliflower (Kalia et al., 2017). This was the first report of SCAR markers found to be tightly linked to black rot resistance locus (*Xca1Bo*) in cauliflower. Interestingly, these markers showed 100% accuracy in differentiating the resistant and susceptible plants of cauliflower breeding lines. Very recently, the SCAR marker, ScOPO-04833, was used as a foreground marker to introgress the black rot-resistance gene (*Xca1bo*) during marker-assisted pyramiding of black rot-resistance gene *Xca1bo* and downy mildew-resistance gene *Ppa3* in popular early cauliflower variety Pusa Meghna (Saha et al., 2021).

**TABLE 1:** List of quantitative trait loci (QTLs)/R-genes associated with black rot resistance in *Brassica oleracea* and related *Brassica* species.

Disease	Species	Cultivar	Mapping population	*Xcc* race	Gene locus/QTL	Chr/LG	Linked marker	References
Black rot *Xanthomonas campestris* pv. *campestris* (Pammel) Dowson	*Brassica oleracea*	BI-16 (resistant) × OSU Cr-7 (susceptible)	F3	–	–	LG1, LG2, LG9	wg2g11, wg6g5, wg6g5, wg1e3b, ec5e12, ec2h2, wg6h1, tg4d2b, wg8a9b, wg4d7, ec2d9, wg8a9b	Camargo et al., 1995
	*Brassica oleracea*	11B-1-12 (resistant) × Snow Ball (susceptible)	F2	4	–	–	OPAB04, UBC 72, UBC 322, UBC 66, UBC 205, UBC 121 UBC 320, UBC 327	Tonguç et al., 2003
	*Brassica oleracea var. capitata*	January King (resistant) × Golden Acre (susceptible)	F2	–	–	–	C-11_1000_	Kaur et al., 2009
	*Brassica oleracea*	GC P09 (susceptible) × Reiho P01(resistant)	F2:3	1	–	LG2, LG3, LG7, LG9	CAM1, GSA1, F12-R12e, BORED, CHI, ASB1, IPI, FLC3	Doullah et al., 2011
	*Brassica oleracea*	CY (resistant) × BB (susceptible)	F2	1	*QTL-1, QTL-2, QTL-3*	C02, C04, C05	BoCL3135s, BoCL5545s, BoCL5989s, BoCL4802s, BoCL4271s, BoCL2635s, BoCL908s, BoCL5694s	Kifuji et al., 2013
	*Brassica oleracea*	GC P09 (susceptible) × Reiho P01 (tolerant)	F2	1	*XccBo(Reiho)2, XccBo(Reiho)1*, *XccBo(GC)1*	C08, C05, C09	BoGMS1330, BoGMS0971, CB10459	Tonu et al., 2013
	*Brassica oleracea* var. *botrytis*	Pusa Himjyoti (susceptible) × BR-161 (resistant)	F2	1	–	–	OPO-04_833_, OPAW-202_538_, OPG-25_625_	Saha et al., 2014a
	*Brassica oleracea* var. *botrytis*	Pusa Himjyoti (susceptible) × BR-161 (resistant)	F2	1	*Xca1bo*	C03	RAPD 04_833,_ ISSR 11_635_	Saha et al., 2014b
	*Brassica oleracea*	C1184 (susceptible) × C1234 (resistant)	F2:3	-	*BRQTL-C1_1, BRQTL-C1_2, BRQTL-C3, BRQTL-C6*	C01, C03, C06	H073E22-3, BoRSdcaps1-11, BoEdcaps4, BoESSR089, BoESSR291, BoRSdcaps3-12, BoRSdcaps1-13, BoRSdcaps1-14, sR12387, BnGMS353	Lee et al., 2015
	*Brassica oleracea* var. *botrytis*	Pusa Himjyoti (susceptible) × BR-161 (Resistant)	F2	1	*Xca1bo*	–	ScOPO-04_833,_ ScPKPS-11_635_	Kalia et al., 2017
	*Brassica oleracea*	TO1000DH3 × Early Big	DH	1	*Xcc1.1, Xcc6.1, Xcc8.1, Xcc9.1*	LG1 (C01), LG6 (C06), LG8 (C08), LG9 (C09)	–	Iglesias-Bernabé et al., 2019
	*Brassica oleracea*	Twenty-seven different cabbage inbred lines	Inbred lines	1, 2, 3, 4, 5, 6, 7	–	C01, C03, C06, C08	BnGMS301, BoESSR726, BoESSR291, OI10G06, BoGMS0971	Afrin et al., 2018a
	*Brassica rapa*	–	F2	4	*R4*	–	WE22, WE49	Ignatov et al., 2000
	*Brassica rapa*	R-o-18 (susceptible) × B162 (resistant)	F2	1	*XccR1d-1, XccR1i-1*	A06	E11M50_280b, E12M48_171r	Soengas et al., 2007
	*Brassica rapa*	R-o-18 (susceptible) × B162 (resistant)	F2	4	*XccR4d-1, XccR4i-1*	A06	E12M61_215b, E12M61_215b	Soengas et al., 2007
	*Brassica rapa*	R-o-18 (susceptible) × B162 (resistant)	F2	4	*XccR4i-2*	A02	E11M59_178r	Soengas et al., 2007
	*Brassica rapa*	R-o-18 (susceptible) × B162 (resistant)	F2	4	*XccR4i-3*	A09	E12M48_1 > 330b	Soengas et al., 2007
	*Brassica rapa*	175 × P143 and P115 × 143	DH	1, 3, 4, 6	13 *QTL* (DH30), 19 *QTL* (DH38)	A01, A02, A03, A04, A05, A06, A07, A08, A09, A10	Many	Artemyeva et al., 2018
	*Brassica carinata*	NPC-17 (susceptible) × NPC-9 (resistant)	F2	1	*Xca1bc*	LG7 (B genome)	At1g70610, At1g71865, Na14-G02	Sharma et al., 2016
	*Brassica napus*	N-o-9 (susceptible) × N-o-1 (resistant)	DH	4	*Xca4*	N5 (A genome)	–	Vicente et al., 2002

All the above researchers reported the simple genetic control or qualitative resistance against *Xcc*1 which is desirable for effective production of black rot resistant hybrids. *Xcc* infects the plants mainly through hydathodes and colonizes the epitheme and kills the host cell by degrading the cell walls. According to Bae et al. (2015), the rapid destruction of epitheme cell may cause the expression of any R-gene expression ineffective. In rice, the R-genes have lost their qualitative feature against a virulent strain of *Xanthomonas oryzae* pv. *oryzae* and had adopted a new, intermediate resistance phenotype (Li et al., 1999). This signifies the importance of quantitative resistance against *Xcc*, and several authors have shown that resistance to *Xcc*1 is quantitative and under polygenic control. Doullah et al. (2011) mapped the QTLs controlling resistance to *Xcc* (later revealed as race 1 by Tonu et al., 2013) and identified two significant QTLs on LG2 and LG9 in *B. oleracea*. These QTLs were compared with already identified QTLs by Camargo et al. (1995). Interestingly, the QTL on LG9 corresponded to the QTL interval (between *wg6g5-wg2g11*) on LG 1 identified by Camargo et al. (1995) enhancing its utility in marker-assisted selection for black rot resistance. Again Tonu et al. (2013) analysed the *Xcc* 1 resistance QTLs in *B. oleracea* by improving an F_2_ population map developed by Doullah et al. (2011) and carried out comparative analysis of the mapped QTLs using common markers. This has led to the development of common markers (pW, pX, and BoCL) closely linked with the previously reported QTLs and could be used as anchor markers to compare the map position of *Xcc*1 resistance QTLs. The authors obtained two major QTLs: *XccBo*(*Reiho*)2 and *XccBo*(*GC*)1, and one minor QTL: *XccBo*(*Reiho*)1 on chromosome C8, C9, and C5, respectively. Based on the common markers, the QTL *XccBo*(*Reiho*)1 corresponded to QTL-LG2a and QTL-3, identified by Camargo et al. (1995) and Kifuji et al. (2013), respectively. Kifuji et al. (2013) mapped one major QTL (*QTL-1*) for *Xcc*1 resistance on linkage group C2 in two consecutive years explaining 15.05 and 9.88% of phenotypic variance, respectively. Two minor QTLs, *QTL-2* (LG C4) and *QTL-3* (LG C5), were also reported. Interestingly, the *QTL-1* region showed synteny with a region spanning from 5.3 to 7.4 Mb on the short arm end of chromosome 5 of *Arabidopsis thaliana*, which was rich in genes of TIR-NBS-LRR family. In another study, dCAPS markers developed from candidate SNPs were used to improve the resolution of a previously developed genetic map and QTL analysis identified one major (*BRQTL-C1_2*) and three minor QTLs (*BRQTL-C1_1*, *BRQTL-C3*, and *BRQTL-C6*) containing 21 candidate resistance genes (Lee et al., 2015).

The most comprehensive study to dissect quantitative resistance to *Xcc1* was carried out by Iglesias-Bernabé et al. (2019). The authors measured five traits, such as initial stages of invasion, success of infection, and spread of the pathogen, in the BolTBDH mapping population and identified four single-trait QTLs (*Xcc1.1*, *Xcc6.1*, *Xcc8.1*, *Xcc9.1*) on linkage group 1, 6, 8, and 9 confirming the quantitative nature of *Xcc*1 resistance as reported by the previous authors. Three QTLs, except *Xcc9.1*, were identified previously. Multi-trait QTL analysis revealed that the spread of *Xcc* is related to the size of the leaf. Two resistance strategies were followed by the genotypes of the mapping population to keep up with the disease progression; reducing the lesion size or maintaining more area of the leaf with photosynthetic activity to be more tolerant to *Xcc* invasion. Also, the authors showed that the resistance mechanisms contributing to variation of resistance could be related to different aspects of plant immunity, including the synthesis of glucosinolates (GSLs) and phenolics.

Compared to *Xcc*1, few studies were conducted to identify QTLs for *Xcc*4 in *B. oleracea*. As the resistance to races 4 is scanty in *B. oleracea*, a resistant line (11B-1-12) was developed by transferring black rot resistance from B-genome of *B. carinata* (provides complete protection against races 1 and 4 of *Xcc*) to *B. oleracea* by protoplast fusion (Hansen and Earle, 1995). This resistance line (11B-1-12) was used to develop three F_2_ populations in *B. oleracea* and eight polymorphic RAPD markers were found linked with completely black rot (*Xcc*4) free plants (Tonguç et al., 2003). The segregation pattern of the linked markers suggested the role of a single dominant major gene governing resistance to *Xcc* 4.

There is a dearth of durable resistance sources in the ‘C’ genome of *B. oleracea* for different *Xcc* races (Soengas et al., 2007). However, ‘A’ and ‘B’ genomes of *Brassica* species are the sources of resistance genes, and several QTLs conferring resistance to black rot were mapped in the related *Brassica* species (Table 1). In *B. rapa* (‘A’ genome), both race-specific and broad-spectrum resistance against six races have been frequently observed. Soengas et al. (2007) identified four highly significant QTLs (two for race 1 and two for race 4) on chromosome A06 using an F_2_ mapping population of 114 plants. Two additional QTLs for resistance to race 4 were found on linkage group A02 and A09. The authors opined that the markers closely linked to the QTLs may assist in the transfer of resistance into different cultivars of *B. oleracea*. Recently, Sharma et al. (2017), while exploring the ‘A’ and ‘B’ genomes of *Brassica* species to transfer black rot resistance into cauliflower, introgressed a single dominant black rot resistance gene, *Xca1bc*, through interspecific hybridisation between cauliflower (Pusa Sharad) and *Brassica carinata* (NPC-9), followed by embryo rescue. A marker ‘At1g70610’ linked with resistance against *Xcc* race 1 (Sharma et al., 2016) was used to confirm the successful introgression of black rot resistance in the interspecific BC1 population.

Recently, Afrin et al. (2018a) screened 27 inbred lines resistant to different races of black rot (1, 2, 3, 4, 5, 6, 7) using 9 simple sequence repeats (SSRs) and 1 insertiondeletions (inDels) markers, and based on the bioassay and molecular screening results, five markers were selected capable of distinguishing the resistant lines from the susceptible ones of cabbage consistently.

Often, loci for black rot resistance identified in green house screening are not detected under field conditions. This could be due to greater experimental error in the field experiments and lower resolution of visual rating scale during field scoring (Horsfall and Cowling, 1978; Jamwal and Sharma, 1986). In this backdrop, Camargo et al. (1995) mapped the QTLs controlling resistance to *Xcc* in field, glasshouse, and genomic regions were identified on LG 1 and 9 associated with both young and adult plant resistance and two additional QTLs (*QTL-LG2a*, *QTL-LG2b*) on LG2 were associated with the young plant resistance. The results mostly indicated that plants selected based on young plant screening should reflect the adult-plant resistance.

In summary, we observed that several QTLs, both with major and minor effects, have been mapped to different chromosomes of *B. oleracea* suggesting that the resistance to black rot is a complex trait. Furthermore, though several QTL mappings were conducted in both *B. oleracea* and related *Brassica* species, so far, no resistance gene has been cloned. Basically, R-gene-mediated effector-triggered immunity (ETI) is considered as the most effective in conferring resistance to plants (Debieu et al., 2016). However, ETI fails to provide durable and broad-spectrum resistance shifting the attention toward quantitative resistance. In *B. oleracea*, black rot resistance is mostly considered to be under quantitative control. However, with the available information of markers/QTLs for black rot resistance, we can say that the information is limited, especially QTLs imparting resistance to race 4 of *Xcc* need to be identified. Also, emergence of new races of *Xcc* is a major factor hindering the deployment of the QTLs in resistance breeding of *Xcc*.

Nevertheless, all the above information of markers/QTLs identified in *B. oleracea* will help in the understanding of the molecular mechanisms of disease response in *B. oleracea* under *Xcc* stress. Introgression of both race-specific and race non-specific genes into the background of susceptible *B. oleracea* cultivars could help in conferring broad-spectrum resistance. Also, the information about the QTLs and linked molecular markers will undoubtedly aid in the introgression of resistance into the elite cultivars of *B. oleracea* to develop resistant varieties.

#### Identification of Candidate Nucleotide-Binding Site-Leucine-Rich Repeat Encoding R genes for Black Rot Resistance

**FIGURE 3:**
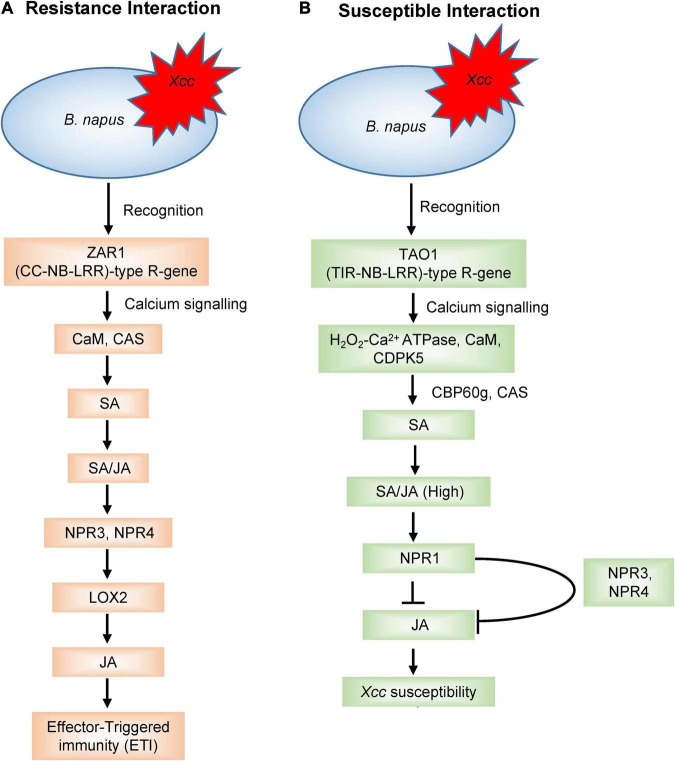
A model outlining the R-gene-mediated signalling to **(A)** induce effector-triggered immunity (ETI) and **(B)** disease susceptibility upon *Xcc* inoculation in *Brassica napus* as proposed by Mamun et al. (2020). JA, jasmonic acid; SA, salicylic acid; CaM, calmodulin; CAS, calcium-sensing receptor; NPR1, non-expressor of pathogenesis-related gene 1; NPR3, non-expressor of pathogenesis-related genes 3; NPR4, non-expressor of pathogenesis-related genes 4; LOX2, lipoxygenase 2; CDPK5, calcium-dependent protein kinase 5; CBP60g, calcium-sensing protein 60 g.

Plants defend themselves from a variety of microbial pathogens by employing two types of resistance: qualitative and quantitative resistance. Qualitative resistance is governed by R-gene-mediated defence where R-genes convey disease resistance by producing R proteins against the plant pathogens. Upon pathogen attack, the phytopathogens produce certain molecules called ‘effectors,’ encoded by Avr (avirulence) genes which are recognised by R-genes and activate the effector-triggered immunity (ETI). This interaction is also known as “gene-for-gene” model for plant disease resistance (Flor, 1971), and such types of interactions have been observed between the *avr* genes (*A1*–*A5*) of *Xcc* and the corresponding *R*-genes (*R1–R5*) of several *Brassica* cultivars (Fargier and Manceau, 2007; Vicente and Holub, 2013). The main class of the R-genes consists of nucleotide-binding site-leucine-rich repeat (NBS-LRR) proteins (Van der Biezen and Jones, 1998; Dangl and Jones, 2001; Yu et al., 2014) and are widely distributed in plants. While the nucleotide-binding site (NBS) domain can bind and hydrolyse ATP/GTP, the leucine-rich repeat (LRR) domain is involved in protein-protein interactions (Tameling et al., 2002; Wan et al., 2012). Based on N-terminal structures, the NBS-LRR type R-genes were further subdivided into coiled-coil-nucleotide-binding site-leucine-rich repeat (CC-NB-LRR) type and toll/interleukin-1 receptor-nucleotide-binding site-leucine-rich repeat (TIR-NB-LRR) type (Dangl and Jones, 2001). Izzah et al. (2014) identified 29 expressed sequenced tags (ESTs) containing NBS-LRR domains, among which, 22 were TIR-NBS-LRRs and 7 were CC-NBS-LLRs type in the black rot resistant cabbage line C1234. Lee et al. (2015) reported 21 different NBS-LRR genes within four resistance QTL regions against *Xcc* in cabbage. Of the detected 21 R-genes, nine were present in gene clusters. Eight NBS-LRR encoding genes were identified in the *BRQTL-C1_1* and *BRQTL-C1_2* QTLs, seven and five NBS-LRR type R-genes were detected near the *BRQTL-C3* and *BRQTL-C6* region, respectively. Importantly, comparison of these 21 candidate genes against *Brassica* database showed the sequence similarity to disease resistance proteins. In another study, DNA sequence variation and expression of 31 NBS-encoding genes were analysed in cabbage, which encoded TIR, NBS, LRR, and RPW8 protein domains and nine NBS-encoding R-genes (Bol003711, Bol010135, Bol010559, Bol022784, Bol029866, Bol042121, Bol031422, Bol040045, and Bol042095) were identified presumed to be involved in black rot resistance (Afrin et al., 2018b). NBS-LRR genes, after recognizing the pathogen, triggers various defence signal transductions leading to hypersensitive response (Sagi et al., 2017). These R-genes regulate phytohormone signalling to counteract the pathogenic infection (Joshi and Nayak, 2011). During host-pathogen interactions, different calcium signalling genes regulate the plant defence (Tortosa et al., 2019). Mamun et al. (2020) hypothesized that R-genes were involved in calcium signalling and hormonal regulation in triggering ETI response and disease susceptibility in the *B. napus*–*Xcc* pathosystem (Figures 3A,B). The expression analysis of R-genes, *ZAR1* (CC-NB-LRR-type) and *TAO1* (TIR-NB-LRR-type), in two contrasting genotypes of *B. napus* revealed that *ZAR1* was involved in the resistance interaction through calcium-sensing receptor (CAS) and calmodulin (CaM) to initiate salicylic acid (SA) synthesis and signalling, thereby inducing JA synthesis and signalling, and resulting in ETI response. On the contrary, *TAO1* mediated the SA accumulation through calcium-sensing receptor (CAS) and calcium-sensing protein 60g (CBP60g), with an antagonistic depression of JA leading to disease susceptibility.

However, ETI often fails to deliver durable and broad-spectrum resistance if the trait is governed by polygenic resistance, shifting the focus toward resistance QTLs. As discussed earlier, resistance to *Xcc* in most of the *B. oleracea* lines was disclosed to be under quantitative control. The durability of quantitative resistance could happen due to the exertion of a low selection pressure on the pathogen population and difficulties to overcome the combination of different resistance-associated mechanisms by the pathogens (Palloix et al., 2009; Mundt, 2014). Resistance QTLs were also reported to be specific or non-specific to a pathogen, and some QTLs can show resistance to multiple pathogens (Ellis et al., 2014; Wiesner-Hanks and Nelson, 2016). In several systems, combination of both broad-spectrum and isolate-specific QTLs resulting in quantitative resistance have been reported (Caranta et al., 1997; Calenge et al., 2004; Rocherieux et al., 2004). The genes accounting for quantitative resistance represent a broad range of possible functions for the genes underlying resistance QTLs, such as basal defence, detoxification, transduction of defence signals, or partially altered major R-genes (Niks et al., 2015; French et al., 2016). In *Arabidopsis thaliana*, *Resistance related KinaSe1* (*RKS1*) conferred quantitative resistance against the races 1, 3, 5, 7, and 9 of *Xcc* (Huard-Chauveau et al., 2013). *RKS1* encodes an atypical kinase lacking the critical domains required for kinase catalytic core during catalysis (Roux et al., 2014). Recently, Debieu et al. (2016) identified two major QTLs that conferred resistance specifically to races 2 and 6 of *Xcc*. The study revealed that the quantitative disease resistance to race 6 involves the well-known immune receptor pair RRS1/RPS4. In addition to *RKS1*, three genes with different range of specificity were involved in conferring resistance to *Xcc*, which suggested that quantitative disease resistance to *Xcc* is governed by a complex network by interconnecting multiple response pathways induced by distinct pathogen molecular determinants (Debieu et al., 2016).

Secondary metabolites like glucosinolates (GSLs) are also involved in quantitative resistance against *Xcc* in *B. oleracea* (Iglesias-Bernabé et al., 2019). *B*. *oleracea* is known for its high content of GSLs whose hydrolysed products have been proven toxic to pathogens. GSLs play an important role in plant defence against *Xcc*, and many workers have described the potential role of GSL in defence against *Xcc* in *B. oleracea* and related *Brassica* species (Aires et al., 2011; Velasco et al., 2013; Madloo et al., 2019; Rubel et al., 2020) which will be discussed later. While dissecting the quantitative resistance against race 1 of *Xcc*, multi-trait QTL analysis identified four QTLs of resistance and the spread of *Xcc* was found related to the size of the leaf (Iglesias-Bernabé et al., 2019). The mechanism of resistance was found to be related with the synthesis of GSLs and phenolics.

However, quantitative resistance governed by minor-effect QTL are difficult to exploit than the major R-genes. In the post-genomic era, improved tools and methods are becoming handy to better integrate the quantitative resistance into plant breeding techniques. In the best scenario, combining major R-genes with quantitative resistance could be proved as an interesting strategy for effective breeding to confer durable resistance to *Xcc* in *B. oleracea*.

### Transcriptomics

Transcriptomics refers to the study of the entire set of RNA of an organism, including mRNAs and other non-coding RNAs (McGettigan, 2013). Transcriptome study helps in characterizing and quantifying the entire RNA present in an organ, tissue, or cell in a given organism. Different genes of a cell are up or downregulated in different physiological and developmental process. So, comparing of RNA expression profile provides an insight into when and where the genes are expressed under different treatments and helps in validation of the putatively differentially expressed genes. This, in turn, helps in the identification of candidate genes influencing any important traits involved in the cellular process of an organism. Transcriptome is highly dynamic unlike the genome which basically remains constant irrespective of age, organ, or growth conditions (El-Metwally et al., 2014). RNA-seq has largely replaced the earlier commonly used approaches for gene expression profiling such as microarray and serial analysis of gene expression (SAGE). Transcriptome analysis reveals the molecular mechanisms underlying specific biological processes and pathogenesis, providing new guidance in disease control and crop improvement (Kell and Oliver, 2016). Additionally, transcriptome studies decipher many dynamic changes occurring in molecular communication during the plant infection by the pathogens. The recent advancement of NGS technologies have allowed the transcriptome sequencing through cDNA sequencing on a massive scale (Voelkerding et al., 2010). This has permitted the researchers to design large-scale experiments to capture and enumerate the transcripts and analyse the transcriptional responses of *B. oleracea* to black rot infection which revealed the role of different genes involved in *B. oleracea-Xcc* interaction (Tables 2, 3).

**TABLE 2:** Published transcriptomic studies in *Brassica* species on black rot resistance.

Species	Plant organ	Time point (Tissue collection)	Methodology	Objective	Inference	References
*Brassica oleracea* var. *capitata*	Leaves	–	RNA-Seq	Identification of ESTs related to the NBS-LRR domain in the black-rot resistant line C1234	29 ESTs containing NBS-LRR domains were identified in the black rot resistant cabbage line C1234, among which 22 were TIR-NBS-LRRs and 7 were CC-NBS-LLRs type	Izzah et al., 2014
*Brassica oleracea*	Leaves	3- and 12-days post-inoculation (dpi)	RNA-Seq	Investigation of molecular changes produced in *B. oleracea* plants infected by *Xcc*	Genes related to terpenes, flavonoids, alkaloids and anthocyanins and phytohormones were up-regulated at early stage of infection	Tortosa et al., 2018a
*Brassica oleracea* var. *capitata*	Leaves, root, silique, and stem	–	RNA-Seq	To identify NBS-encoding genes linked to black rot resistance in cabbage	31 NBS- genes encoding TIR, NBS, LRR and RPW8 protein domains were differentially expressed in leaves, root, silique and stem tissues of cabbage. Several of these genes were highly expressed in resistant compared to susceptible cabbage lines.	Afrin et al., 2018b
*Brassica oleracea* var. *italica*	Leaves	3- and 12-days post inoculation (dpi)	RNA-Seq	To investigate the transcriptome dynamics of *Brassica oleracea* in response to *Xcc* race 1	Two calcium-signalling proteins (CBP60g and SARD1) regulated the plant transcriptomic response in the resistance against *Xcc* which was confirmed using Arabidopsis knockout mutants	Tortosa et al., 2019
*Brassica oleracea* var. *capitata*	Leaves	0- and 6-days after *Xcc* inoculation	RNA-Seq	Genome-wide identification, expression profile of the TIFY gene family in *B. oleracea* var. *capitata*, and their response to various pathogen infections including *Xcc*	36 TIFY genes were identified including 22 JAZ genes and the JAZs were induced and inhibited after *Xcc* infection in the resistance line, indicating their probably distinct roles in disease resistance or susceptibility.	Liu et al., 2020
*Brassica oleracea*	Leaves	0-, 12-, 24-, 48- and 96-h post-inoculation (hpi)	RNA-Seq	Transcriptome analysis of resistant and susceptible lines of *B. oleracea* in response to early infection with *Xcc*	Genes related to glucosinolate biosynthesis and catabolic pathways, ROS scavenging, photosynthetic energy metabolism, hormonal receptor-kinase-related genes and NBS-encoding resistance genes were enhanced during the early infection period.	Sun et al., 2020
*Brassica oleracea* var. *capitata*	Leaves	3-days after inoculation	RNA-Seq	Transcriptomic analysis of resistant and susceptible cabbage lines to decipher the molecular bases and mechanisms of early-phase response against black rot	Top ten differential expression genes were found to contain NBS-LRR genes, protein kinase genes and expansin genes indicating some genes playing key roles in the regulation of early response to black rot infection.	Song et al., 2020

**TABLE 3:** List of differential gene expression studies in *Brassica* species on black rot resistance.

Species	Plant organ	Time point (Tissue collection)	Objective	Inference	References
*Brassica oleracea* var. *botrytis*	Leaves	–	Cloning of differentially expressed fragments in cauliflower after *Xcc* inoculation	M6 gene fragment was identified as a new H_2_O_2_ downstream defence related gene fragment which could be induced by *Xcc* and H_2_O_2_.	Gu et al., 2008
*Brassica oleracea* var. *botrytis*	Leaves	0-, 2-, 6-, 12-, 24-, 48-, 72-, and 96-h post- infection	Identification of differentially expressed genes associated with resistance to *Xcc* in cauliflower	Gene expression of 12 genes corresponding to a range of functional categories including metabolism, photosynthesis as well as cell defense (plant defensin gene *PDF1.2*, lipid transfer protein, thioredoxin h) in response to *Xcc* was quicker and more intense in cauliflower resistant line C712 suggesting their involvement in the response against *Xcc* infection.	Jiang et al., 2011
*Brassica* *oleracea* var. *capitata*	Leaves	12-, 24-, and 48-h post- inoculation	To identify genes involved in resistance mechanisms against *Xcc* in cabbage	A total of 150 unigenes obtained were classified into five major functional categories: metabolism, disease and defence-related, structural proteins, signalling pathway related and unclassified group. The defence-specific genes showed increased expression in the resistant cultivar and elicited a strong hypersensitive response upon attack by black rot.	Roohie and Umesha, 2015
*Brassica rapa* var. *glabra*	Leaves	1-, 2-, 3-days post-inoculation (dpi)	Differential defence responses of susceptible and resistant kimchi cabbage cultivars to black rot	*PR1*, *BGL2*, *Chi1*, *PR4*, *VSP2*, *LOX2* and *GST1* were differentially regulated in the kimchi cabbage leaves during resistance reaction and the resistance was strongly associated with the hormone dependent transcriptional induction of defence genes.	Lee and Hong, 2015
*Brassica oleracea*	Leaves	24 h after inoculation	Role of microRNAs (miRNAs) in *B. oleracea* resistance against *Xcc*	The decreased expression of miR156, miR169 and miR390 may be involved in a stress-induced flowering phenomenon due to *Xcc* infection. miR167, as miR390, modulates the expression of auxin response factors (ARFs) and may be involved in a PAMP-triggered immunity response. The upregulation of the 4 miRNAs could play a role in *B. oleracea* resistance enhancement against *Xcc*.	Santos L.S. et al., 2019
*Brassica napus*	Leaves	14-days post- inoculation	To elucidate the cultivar variation in disease susceptibility and disease responses in relation to hormonal status in the interaction of *Brassica napus* cultivars and *Xcc*	The ratios of ABA/JA and SA/JA increased with enhanced expression of SA signalling regulatory gene (*NPR1*) and transcriptional factor (*TGA1*) with antagonistic suppression of JA- regulated gene *PDF 1.2*. In the resistant cultivar, defensive metabolites accumulated with the enhanced expression of genes involved in flavonoids (chalcone synthase), proanthocyanidins (anthocyanidin reductase), and hydroxycinnamic acids (ferulate-5-hydroxylase) biosynthesis and higher redox status were observed, whereas the opposite results were obtained for susceptible cultivars.	Islam M.T. et al., 2017
*Brassica napus*	Leaves	14-days post- inoculation	To investigate the hormonal regulations in soluble and cell wall-bound phenolic compound accumulation in the resistant and susceptible cultivar of *Brassica napus*	Enhanced expression of JA signalling was concurrently based on transcriptional up-regulation of *PAP1*, MYB transcription factor, and phenylpropanoid biosynthesis genes (*CHS*, *F5H*, *COMT1*, and *CAD2*) which induced the higher accumulation of defensive metabolites such as hydroxycinnamic acids and flavonoids in the resistant cultivar.	Islam et al., 2019b
*Brassica oleracea* var. *capitata*	Leaves	1-, 3-, and 5-days after inoculation (DAI)	To understand the role of glucosinolate biosynthesis and breakdown-related genes for resistance against *Xcc* in cabbage	Positive and significant association between aliphatic GSL compounds and expression values of transcription factor and GSL biosynthesis-related genes (*ST5c-Bol030757* and *AOP2-Bo9g006240*) as well as between indolic GSL compounds and the expression of transcription factor and GSL biosynthesis-related genes (*MYB34-Bol017062*, *MYB122-Bol026204*, *CYP81F2-Bol012237*, *CYP81F4-Bol032712* and *CYP81F4-Bol032714*) were reported.	Rubel et al., 2020
*Brassica napus*	Leaves	14-days post- inoculation (DPI)	To investigate the involvement of R-gene-mediated calcium signalling and hormonal signalling in Effector-triggered immunity (ETI) or susceptibility in the *Xcc–B. napus* pathosystem	In the resistance interaction (ETI), R-gene (*ZAR1*) and related genes (*NDR1, MAPK6*), SA receptor, (*NPR3* and *NPR4*), JA synthesis (*LOX2*) and signalling (*PDF1.2*) genes were up-regulated while calcium signalling-related genes (*Ca2CATPase, CDPK5, CBP60g*) were down-regulated. In the susceptible interaction, R-gene (*TAO1*), SA synthesis (*ICS1*) and signalling (*NPR1*), calcium-signalling-related genes (*Ca2CATPase, CDPK5, CBP60g*), SA synthesis (*ICS1*) genes were up-regulated whereas JA synthesis (*LOX2*) and resistance related gene (*MAPK6*) were down-regulated.	Mamun et al., 2020

Over the years, transcriptome analysis has been used to understand the plant–microbe interactions (Wulf et al., 2003). Gene regulation studies in response to pathogen attack may indicate the role of the relevant defence genes (Singh et al., 2018). Suppression subtractive hybridisation (SSH) is regarded as a powerful approach for identification of differentially expressed genes including the response of the plants to pathogen infection (Jin et al., 2010; Luo et al., 2010; Yue et al., 2010) which could help in the global analysis of gene expression. SSH does not require sequence information to study differential genes (Kathju et al., 2006) and is often used to identify the genes responding to pathogens and stresses in plants (Xiong et al., 2001). So, Jiang et al. (2011) attempted to understand the molecular mechanisms of resistance of cauliflower in response to *Xcc* infection combining SSH with RT-PCR. An SSH cDNA library comprising many defence-related genes including plant defensin gene *PDF1.2*, lipid transfer protein, thioredoxin h., etc. was established and 12 differentially expressed genes associated with *Xcc* resistance were identified. Roohie and Umesha (2015) also employed the SSH technique to identify the genes involved in black rot resistance mechanisms in *B*. *oleracea* var. *capitata*. Out of 150 unigenes (classified in to five functional categories), 35% of the unigenes accounted for the defence-related unigenes. Defence-specific representation of the genes was confirmed by semi-quantitative RT-PCR and its increased expression in the resistant cultivar was validated by qPCR.

Several researchers have reported the differential responses of genes in the susceptible and resistant cultivars of *B. oleracea* to black rot disease (Table 3). Cloning of differentially expressed cDNA fragments obtained from black rot resistant cauliflower plants revealed that M6 gene fragment was a new H_2_O_2_ downstream defence-related gene fragment and could be induced during infection by *Xcc* (Gu et al., 2008). Transcriptomic analysis of the leaves of *B. oleracea* collected 3 and 12 dpi revealed the up-regulation of 78 and 809 genes and a downregulation of 10 and 169 genes in the early and late responses, respectively (Tortosa et al., 2018a). During *Xcc* attack, genes related with terpenes, flavonoids, alkaloids, anthocyanins, SA, ethylene, and JA were up-regulated in early response exhibiting their importance during pathogenesis. To analyse the dynamics of the transcriptional response of *B. oleracea* plants infected with *Xcc*, Tortosa et al. (2019) highlighted the role of Ca^+2^ signalling proteins as secondary messenger for several downstream signalling processes which include the activation of several transcription factors involved in the SA-mediated host defence in *B. oleracea*. Two calcium-signalling proteins (*CBP60g* and *SARD1*) played important roles in resistance against *Xcc* which was, again, confirmed by *Arabidopsis* knockout mutants. In another study, Lee and Hong (2015) analysed the pathogenesis-related (PR) gene expression during resistance and susceptible responses to black rot disease in kimchi cabbage. The semi-quantitative RT-PCR analysis revealed the transcriptional activation of *PR1*, *BGL2*, *Chi1*, *PR4*, *VSP2*, *LOX2*, and *GST1* in the leaves of resistant genotypes as compared to susceptible genotypes. Also, the PR genes were found to be regulated by defence-related hormones such as SA, JA, and ethylene. The results indicated that differential defence signalling crosstalk and PR gene expression are involved in cultivar specific resistance against several fungal diseases including black rot in kimchi cabbage and, importantly, the resistance was strongly associated with the hormone-dependent transcriptional induction of defence genes. Jasmonate and other related signalling compounds are involved in the host immunity of plants (Thines et al., 2007; Melotto et al., 2008). A *B. oleracea LOX* gene was cloned in cabbage which was involved in jasmonic acid biosynthesis, and the quantification of transcript levels showed that *BoLOX* transcripts were accumulated rapidly after various biotic stresses (Zheng et al., 2007). The JASMONATE ZIM-DOMAIN (JAZ) proteins are the key repressors of jasmonate (JA) signalling and plays key role in plant defence responses (Chini et al., 2007; Thatcher et al., 2016). To investigate the expression difference of cabbage JAZ genes after infection with *Xcc*, RNA-seq data of both resistant and susceptible materials were analysed by Liu et al. (2020) and many JAZ genes were found to be up-regulated in both the resistance and susceptible lines.

For better resistance against *Xcc*, it is essential to understand the infection and multiplication process of *Xcc* in host cells, and tremendous progress have been made in this direction with the identification and characterisation of more than 100 genes contributing to *Xcc* virulence (Chan and Goodwin, 1999; He et al., 2007; He and Zhang, 2008; Büttner and Bonas, 2010). Liao et al. (2016) investigated the potential role of *prc* gene in the pathogenicity of the black rot and the transcriptional profiling of the wild type and mutant showed that the mutation of *prc* in *Xcc* leads to the alteration of the transcription level of 91 genes. The genes were associated with a range of biological functions such as carbohydrate transport and metabolism, cell wall/membrane biogenesis, post-translational modification, protein turnover and chaperones, inorganic ion transport, metabolism, and signal transduction mechanisms, providing new information about the regulatory role of *prc* gene. Micro RNAs (miRNAs) are known to be associated with various biological processes including abiotic and biotic stresses (Rubio-Somoza et al., 2009; Kruszka et al., 2012). miRNAs are conserved and are involved in many molecular interaction networks including plant-pathogen interactions (Sunkar et al., 2012; Islam W. et al., 2017). The role of miRNAs in plants was examined in response to *Xcc* infection and four miRNAs (miR156, miR167, miR169, and miR390) were found to be differentially expressed showing a down and up-regulated expression profile in the susceptible and resistant cultivars, respectively (Santos L.S. et al., 2019). This suggested the possible role of miRNAs in enhancing the resistance of *B. oleracea* against *Xcc* and could be used as potential resistance markers for *B. oleracea-Xcc* interaction. Understanding the early response to black rot holds the key to control black rot and reduce the crop losses. To understand the molecular basis of early-phase response of different resistant cabbage lines against black rot infection, a comprehensive transcriptome analysis of resistant and susceptible lines identified 10,030 differentially expressed genes (Song et al., 2020). Three hundred and eighty four differentially expressed genes overlapped in the susceptible and resistant cabbage lines and the top ten genes contained NBS-LRR type, protein kinase, and expansin genes, indicating their role in early response to black rot infection. Again, transcriptome analysis of the leaves of *Xcc*-resistant (QP07) and susceptible (DBP71) lines was carried out to understand the early defence response (Sun et al., 2020). A total of 3,357 up and 4,091 down-regulated genes were identified between QP07 and DBP71 and functional annotation pathway analysis indicated the enhancement of the ROS scavenging, glucosinolate biosynthesis and catabolic pathways, hormonal, receptor kinase-related genes, and (NBS)-encoding R-genes during the early infection period. Furthermore, photosynthetic energy metabolism was found to be actively regulated by the host plant in response to *Xcc* infection. Glucosinolates (GSLs) play important roles in plant defence mechanisms against necrotrophs, biotrophs, and hemibiotrophs and are broadly found in different *Brassica* species. However, the information about GSL-mediated resistance mechanisms and GSL biosynthesis and catalysis related gene expression after black rot infection is limited. In a recent study, Rubel et al. (2020) found positive and significant association between the aliphatic and indolic GSL compounds with the expression values of transcription factor and GSL biosynthesis-related genes in cabbage. The phytohormones, SA and JA, are the central regulators in hormonal signalling pathways to induce defence response against the pathogens by inducing the genes related to phenylpropanoid synthesis pathway which produces an array of defensive metabolites and these genes were differentially expressed for *Xcc* resistance and susceptibility in *Brassica* species. Islam et al. (2019b) demonstrated that the enhanced expression of JA signalling was concurrently based on transcriptional up-regulation of *PAP1*, MYB transcription factor, and phenylpropanoid biosynthetic genes (*CHS*, *F5H*, *COMT1*, *CAD2*) which induced the higher accumulation of defensive metabolites such as hydroxycinnamic acids and flavonoids in the resistant cultivar (cv. Capitol). Another study reported the differential expression of the NBS-LRR encoding R-genes (*ZAR1* and *TAO1*) and related genes (*MAPK6*), calcium signalling-related genes (*Ca2+ATPase*, *CDPK5*, *CBP60g*, *CAS*, *CaM*), SA receptor (*NPR3, NPR4*), synthesis and signalling (*ICS1, NPR1*) genes, JA synthesis (*LOX2*), and signalling (*PDF 1.2*) genes in the contrasting genotypes of *B. napus* which indicated that JA induced an antagonistic depression of SA suggesting the proper maintenance of SA/JA ratio as a part of the resistance mechanisms against *Xcc* (Mamun et al., 2020), also proved by Islam M.T. et al. (2017).

Taken together, it could be inferred that genes related to photosynthesis, glucosinolate biosynthesis and catabolism, phenylpropanoid biosynthesis, ROS scavenging, calcium signalling, hormonal signalling and synthesis pathway, receptor-kinase-related genes, and NBS-encoding resistance genes were differentially expressed upon *Xcc* infection. The examination pattern of hormone-related DEGs revealed that instead of SA signalling pathway, JA signalling pathway may play a critical role in host resistance to hemibiotrophic pathogen such as *Xcc*. The up-regulation of the genes involved in glucosinolate biosynthesis and catabolic processes during early infection confirmed the role of glucosinolate hydrolytic products in the defence of *Brassica* species against *Xcc*. The enhancement of resistance to *Xcc* by GSLs has been confirmed by several proteomic and metabolomic studies. Several transcriptome studies have revealed the NBS-LLR encoded resistance genes as the key regulators involved in black rot resistance in *Brassica* species. Also, the transcriptome studies revealed that photosynthesis is playing a major role in the interaction between *Xcc* and *Brassica* species. Down-regulation of the DEGs involved in photosynthesis in the resistant plants and up-regulation in susceptible plants suggested that the susceptible plants require more energy to cope up with the infection by *Xcc*. In contrast, by reducing the photosynthetic metabolism, the resistant plants try to control the energy supply of *Xcc*, thereby inhibiting its growth. This also shows the greater resilience of the resistant plants in restoring the normal photosynthetic metabolism compared with the susceptible plants. Upon pathogen attack, rapid production of reactive oxygen species (ROS) leading to oxidative burst is described as one of the earliest responses of the host to pathogen infection onsetting the hypersensitive response. However, to adapt to ROS toxicity, the enzymatic and non-enzymatic antioxidants are activated to scavenge the ROS and reduce the oxidative stress which is part of the resistance mechanisms. The up-regulation of the ROS scavenging enzymes such as catalase, superoxide dismutase, glutathione peroxidase 5, and several glutathione S-transferases in the resistant plants indicated that balancing of host oxidative stress response is essential to efficiently control *Xcc*.

### Proteomics

Proteomics is the high-throughput study of total proteins expressed in a particular organism, organ, specific tissue, and cell of an individual in a given time or developmental stage. Proteomics deals with the analysis of protein–protein interactions, protein expression profiles, protein trafficking, localisation, and their various roles in different cellular processes. Unlike the genome of an organism which is relatively fixed, the proteome is highly dynamic similar to transcriptome and changes based on temporal or environmental factors. There are many proteins present in an organism and its presence is dependent on several factors, including the response to abiotic and biotic stress (Renaut et al., 2006). Proteomics is regarded as a tool for functional genomics in plants and serve to analyse major signalling and biochemical pathways and complex responses of plants to environmental stimuli (Setia and Setia, 2008). Protein is the final executors of most of the biological processes translating plethora of genomic information into functional information. Proteomics could be very informative while studying the plant stress response and tolerance either in a genome-wide or sample-scale (Nakagami et al., 2012). Post-transcriptional modifications such as proteolysis, glycosylation, phosphorylation, nitrosylation, and ubiquitination mediate the functions of a large fraction of proteins (Mann and Jensen, 2003; Beck et al., 2006) playing a key role in intracellular signalling, controlling of enzyme activity, protein turnover, transport, cell structure integrity (Wu et al., 2011), and also to understand the molecular mechanisms of plant-pathogen interactions (Quirino et al., 2010; Lodha et al., 2013). Quantitative proteomics could reveal the differentially expressed proteins contributing to stress response process as well (Liu et al., 2015).

Several powerful techniques are used to identify and quantify proteins of complex biological samples. The most widely used methods during high-throughput protein analysis are gel-based techniques (Chevalier, 2010). The quantitative measurement of proteins can be performed using SDS-PAGE, but for identification and characterisation of the separated proteins, two-dimensional polyacrylamide gel electrophoresis (2DPAGE or 2-DE) and mass spectrometry (MS) is required (Eldakak et al., 2013). 2-DE has become the most versatile tool for protein separation as it resolves the proteins based on both isoelectric point (separated according to their pI in pH gradient PAGE) and molecular mass (SDS PAGE, separated according to molecular weight) (Pomastowski and Buszewski, 2014). However, some disadvantages such as labour intensiveness, low reproducibility, insensitiveness to low-copy number proteins, etc. (Eldakak et al., 2013) have hampered the utility of this method. A modified version of 2-DE, difference gel electrophoresis (2D-DIGE), circumvents most of these issues which can control gel-to-gel variation, allows multiple samples to be co-separated, and enhance the reproducibility (Beckett, 2012). Currently, mass spectrometry (MS) is the most commonly used technique for proteome analysis (Aebersold and Mann, 2003). Before MS analysis, the pre-separation of complex protein mixtures is done by 2-DE and cleaved into smaller peptides. Different types of MS methods have enhanced automation in proteome analysis and have replaced the gel-based separation techniques of peptides. Mass spectroscopy includes several approaches such as liquid chromatography–mass spectrometry (LC-MS/MS), ion trap–mass spectrometry (IT-MS), and matrix-assisted laser desorption/ionisation–mass spectrometry (MALDI-MS), etc. (Helmy et al., 2011, 2012; Komatsu et al., 2014; Shao et al., 2015).

Comparative proteome analysis may help in understanding different biotic stresses in *B. oleracea* as the language of plant-pathogen lies in the proteins. After the first proteome analysis carried out in the model plants *Arabidopsis thaliana* (Kamo et al., 1995) and rice (Komatsu and Tanaka, 2005), several advances were made in proteomics to answer the complex biological questions. In *B. oleracea*, proteomic approach was used to understand the mechanisms of interaction of black rot with the host plants (Table 4). Andrade et al. (2008) employed *in vivo* proteome analysis for protein expression characterisation of *Xcc* in close interaction with *B. oleracea* and showed that *in vivo* expression method originally developed for *Xanthomonas axonopodis* pv. *citri* can be successfully employed for *Xcc*. So, for the first time, i*n vivo* global proteome analysis of *Xcc* was carried out and the protein profiles of *Xcc* was compared during the interactions with resistant and susceptible cultivars of *B. oleracea* (Villeth et al., 2009). The results obtained revealed a group of proteins exclusive to the resistance interaction. Interestingly, different isoforms of the same protein were found in the resistance and susceptible interactions, indicating the same protein playing different roles depending on the types of interaction. The authors also observed the up-regulation of proteins involved in photosynthesis during the resistance interaction.

**TABLE 4:** Published proteomic analyses in *Brassica* species during interaction with black rot.

Species	Plant organ	Time point (Tissue collection)	Methodology	Objective	Inference	References
*Brassica oleracea* var. *capitata*	Leaves	24-h after inoculation (hai)	2-DE	To identify the *Brassica oleracea* proteins during early infection by *Xcc*	Peroxiredoxin precursor protein decreased in the susceptible genotype inoculated with *Xcc*. Proteins involved in photosynthesis were also modulated by *Xcc* infection.	Costa et al., 2014
*Brassica oleracea*	Leaves	5-, 10-, and 15- days after inoculation (DAI)	2-DE, MALDI-TOF	To identify the proteins in susceptible and resistant *Brassica oleracea* in response to *Xcc* infection	Susceptible interaction showed a clear reduction in the abundance of proteins involved in energy metabolism and defence whereas in the resistance interaction, these proteins showed an opposite behavior. Resistance was correlated with the ability of the plants to keep sufficient photosynthesis metabolism activity to provide energy supplies necessary for an active defence.	Villeth et al., 2016
*Brassica oleracea*	Leaves	24-h after *Xcc* inoculation (hai)	2-DE, MALDI TOF-TOF	Identification of *Brassica oleracea* resistance-related proteins at an early stage of infection by *Xcc*	Reduction of photosynthesis-related proteins was observed both in the resistance and susceptible interactions. Also, decreased abundance of ubiquitin (to stop the bacteria from using ubiquitination pathway) and malate dehydrogenase (to reduce energy metabolism in the early stage of infection) were found playing important role in the resistance mechanism against *Xcc*.	Ribeiro et al., 2018
*Brassica oleracea*	Leaves	3- and 12-days post-infection	MALDI-TOF-TOF	To investigate the molecular changes at the protein level in *Brassica oleracea* plants infected by *Xcc*	Proteins shared between early and late response were related to photorespiration, calvin cycle and light reactions, and are strongly down-regulated after *Xcc* infection. Proteins related with glucosinolates degradation (myrosinase) were up-regulated in both early and late response. Proteins related with signalling were up-regulated in late response	Tortosa et al., 2018a
*Brassica oleracea* var. *capitata*	Leaves	24-h after infiltration (hai)	LC-MS/MS	Proteomic analysis of cabbage inoculated with *Xcc* and functional validation of *Brassica oleracea* endochitinase involved in resistance to *Xcc*	Differentially abundant proteins were involved in cell metabolism, protein biosynthesis, processing and degradation, photosynthesis and disease/defence response. A CHI-B4 like gene, encoding an endochitinase showed a high increased abundance in resistant *Xcc*-inoculated leaves and was functionally validated in *Arabidopsis thaliana*	Santos C. et al., 2019
*Brassica napus*	Leaves	14-days after inoculation	LC–MS/MS	Quantitative proteomic analysis of susceptible and resistant *Brassica napus* cultivars infected with *Xcc*	All proteins involved in protein degradation and C2 oxidative cycle and glycolysis, innate immunity-related proteins (zinc finger domain (ZFD)-containing protein, glycine-rich RNA-binding protein (GRP) and mitochondrial outer membrane porin), PS I proteins, ATP synthase, and ferredoxin-NADP+ reductase, redox-related proteins were up-regulated in the resistant cultivar (cv. Capitol) whereas Photosystem II-related proteins were down-regulated	Islam et al., 2021
*Brassica oleracea*	Proteome of *Xcc* extracted from leaves of infected *B. oleracea*	0-, 1-, 2-, 4- and 6-days after inoculation (DAI)	2-DE, MALDI-TOF/TOF	To characterize the protein expression of *Xcc* in close interaction with *B. oleracea*	Several proteins expressed in vivo were identified and were related mainly to metabolism.	Andrade et al., 2008
*Brassica oleracea*	Proteome of *Xcc* extracted from leaves of infected B. oleracea	0-, 1-, 2- and 3-days after inoculation (DAI)	2-DE	To analyze the expressed proteins of *Xcc* exclusive to resistance interaction in *B. oleracea*	Protein profile comparison revealed a group of proteins exclusive to the resistance interaction like Rubisco. Upregulation of proteins involved in photosynthesis in the resistance interaction included intact Rubisco subunits and an oxygen-evolving protein. The presence of different isoforms of the same protein in the resistance and the susceptible interactions indicated that the same protein may play different roles depending on the types of interaction.	Villeth et al., 2009
*Brassica oleracea*	Proteome of *Xcc* extracted from leaves of infected *B. oleracea*	0-, 24-, 48-, 72-, and 120-h after inoculation (hai)	2D-nano UPLC/MS	To study the interaction of *B. oleracea*–*Xcc* using an *in vivo* system to identify proteins involved in pathogenicity	Pathogenicity related proteins [acetylornithine (ArgD)] and several defence and stress-related proteins (lipoxygenase, annexins, apocitocrome f, antimicrobial compound phytoalexin) were observed in the susceptible (REK) and resistant (REU) *Brassica* plants, respectively. Also, proteins associated with photosystems were identified in the resistant plants. A model of *Xcc*-susceptible host interaction was proposed and showed that *Xcc* increases the abundance of several crucial proteins for infection and cell protection.	Santos et al., 2017

To identify the proteins involved in pathogenicity, the interaction of *Xcc-B. oleracea* was studied using an *in vivo* system in three conditions using the label free shotgun 2D-nanoUPLC/MSE (Santos et al., 2017). A model for *Xcc*-susceptible host interaction was proposed, which showed that *Xcc* increases the abundance of proteins required for pathogenicity and cell protection. Pathogenicity related proteins such as acetylornithine (ArgD) and several defence and stress-related proteins (lipoxygenase, annexins, apocitocrome f, antimicrobial compound phytoalexin) was observed in the susceptible (REK) and resistant (REU) *Brassica* plants, respectively. Also, proteins associated with photosystems were identified in the resistant plants. Importantly, the confirmation of the differential expression of the selected genes indicated that these genes, directly or indirectly, are involved in the *Xcc* colonisation of the host plant which could be used as future targets for knock-out studies to confirm their role in the pathogenicity. Identification of proteins expressed during plant–pathogen interactions to know which proteins confer disease resistance is essential to understand the plant-pathogen interactions. A novel peroxidase isozyme and lignification in hydathodes were involved in resistance to black rot disease in cabbage (Gay and Tuzun, 2000). Costa et al. (2014) identified 22 differential proteins during early infection by *Xcc* in *B. oleracea*. One of the proteins identified was precursor of peroxiredoxin which was decreased in the susceptible genotype, and proteins involved in the photosynthesis were also found to be modulated by *Xcc* infection which may help in better understanding of the *B. oleracea*–*Xcc* interaction.

A study by Vega-Álvarez et al. (2021) has shown that *Xcc* infection reduces biomass and photosynthesis in the aerial parts of the seedlings though no effect was detected on the leaves or the biomass of the inoculated adult plants of *B. oleracea*. The biochemical studies state that stomatal closure happens in the presence of *Xcc* (Ho et al., 2013). Abscisic acid (ABA) is a signalling molecule which can suppress the plant immune response. It has been shown that increased abundance of proteins involved with ABA may favour susceptibility (Kim et al., 2011; Desclos-Theveniau et al., 2012). The ABA signalling pathway is manipulated by a type III effector (AvrXccC8004) of *Xcc*, thereby increasing the ABA levels (Ho et al., 2013) and proteins responsive to ABA (Santos C. et al., 2019) during the infection by *Xcc*. ABA along with ROS and elicitors of plant defence may stimulate Ca^2+^ influx (Tortosa et al., 2019) which may increase Ca^2+^ in guard cells prompting stomatal closure (Klüsener et al., 2002). So, stomata can work as part of the innate immunity of a plant by preventing *Xcc* entry (Gudesblat et al., 2009).

*Xcc* infection promotes changes in the secondary and primary metabolism in the host to induce defence programs affecting growth and development (Vega-Álvarez et al., 2021). The changes mostly include down-regulation of proteins involved in photosynthesis (Ribeiro et al., 2018; Santos C. et al., 2019). An earlier study (Villeth et al., 2016) had shown a clear reduction in the abundance of proteins involved in energetic metabolism in susceptible interaction with an opposite behaviour in the resistance interaction. This study indicated that resistance to black rot in *B. oleracea* is correlated with the ability of the plants to keep sufficient photosynthesis metabolism activity to provide energy supplies necessary for an active defence. This was also reported by Villeth et al. (2009). Later, detailed studies of photosynthesis-related proteins upon *Xcc* attack in *Brassica* spp. divulged the down-regulation of these proteins in the resistance reactions. Ribeiro et al. (2018) reported the reduction of photosynthesis-related proteins in the susceptible plants of *B. oleracea* at an early stage of infection with *Xcc*. Also, decreased abundance of ubiquitin (to stop the bacteria from using ubiquitination pathway) and malate dehydrogenase (to reduce energy metabolism in the early stage of infection) were found, playing important roles in the resistance mechanisms against *Xcc*. Proteome analysis of *Xcc*-infected young cabbage leaves and chloroplast-enriched samples of both the susceptible and resistant cultivars revealed the differential abundance of photosynthesis-related proteins in both resistance and susceptible interactions (Santos C. et al., 2019). As expected, most of the photosynthesis-related proteins showed decreased abundance (18%) in the resistance interactions, whereas in the susceptible interaction, increased abundance of proteins was observed consistent with the previous result obtained by Ribeiro et al. (2018). Additionally, proteome analysis revealed the differentially abundant proteins involved in cell metabolism, protein biosynthesis, processing and degradation, and disease/defence response. Among the genes encoding differential proteins, the functional validation of a CHI-B4 like gene encoding an endochitinase showed that the transgenic plants were highly resistant to *Xcc* compared to the wild type which may assist the future breeding programs targeting at black rot resistance in *B. oleracea*. Islam et al. (2021) described the down-regulation of photosystem II (PS II)-related proteins in the resistant *B. napus* cultivar (cv. Capitol) while the PS I proteins, ATP synthase, and ferredoxin-NADP^+^ reductase were up-regulated during the characterisation of the resistance mechanisms in the *B. napus*–*Xcc* pathosystem. In the resistant cultivar, the innate immunity-related proteins [Zinc finger SWIM domain-containing 7 isoform X2 (ZFD), glycine-rich RNA-binding GRP1A isoform X1 (GRP1A) proteins and mitochondrial outer membrane porin] were highly enhanced. Also, redox-related proteins (thioredoxin, 2-cys peroxiredoxin, and glutathione S-transferase) were up-regulated in the resistant cultivar with high NADH, ascorbate, and glutathione-based reducing potential. However, the proteins mostly involved in protein degradation, C2 oxidative cycle and glycolysis were highly activated in the susceptible cultivar (cv. Mosa).

In summary, though comparative proteomic analysis is an efficient and powerful approach to understand the defence mechanisms of *Xcc*–*Brassica* pathosystem, proteomic studies are still at the infancy stage except a handful of works unlike the genomic and transcriptome studies. Nevertheless, the proteomic analysis identified proteins related to photosynthesis, energy metabolism, innate immunity, ROS production and proteolysis, redox homeostasis, and defence signalling pathways involved in the *Xcc*–*Brassica* interaction. Specifically, most of the studies focused on the regulation of the photosynthesis-related proteins as a resistance response in both early and later stages of infection. It is suggested that in *Brassica*, during resistance interactions, photosynthesis is increased at later stages of infection while it is down-regulated at early stages. So, it was hypothesized that the plant tries to minimize the damage caused by *Xcc* by regulating photosynthesis-related proteins. Additionally, decreased abundance of ubiquitin and of malate dehydrogenase was hypothesized to play an important role in the resistance mechanisms against *Xcc*. ZFD and GRP1A proteins were up-regulated in the resistant plants and could be the key regulators of ETI driven innate immunity against *Xcc* in *Brassica* species. Also, R-protein-mediated mitochondrial permeability transition (MPT) enhanced the accumulation of mitochondrial outer membrane porin triggering programmed cell death, thereby inducing resistance to *Xcc*.

### Metabolomics

Metabolome is the complete set of metabolites present in an organism, organ, tissue, and metabolomics refer to the comprehensive profiling of the metabolome of an organism in a particular moment. Metabolomics lies at the phenotypic end of the omics spectrum, capturing the results beginning with the genome and progressing through the transcriptome and proteome (Liu and Locasale, 2017). Metabolomics is the newest among the ‘-omic’ spectrum and has a broad field to develop. Generally, metabolomics is used in combination with transcriptomics or proteomics to investigate the correlation between metabolite levels and genes or protein expression level (Srivastava et al., 2013). The metabolome mainly consists of primary metabolites (involved in the basic functions of the living cell) and secondary metabolites (play important role in plant defence against pests and diseases, Verpoorte et al., 2007). The field of metabolomics has rapidly grown in the past two decades thanks to the advancement in analytical methods and data analysis allowing the understanding of a vast diversity of metabolites within a given sample. Among several state-of-the-art analytical instruments and separation technologies, nuclear magnetic resonance (NMR), gas/liquid chromatography coupled with mass spectrometry (GC-MS/LC-MS), and capillary electrophoresis/mass spectrometry (CE-MS) are the most widely used tools (Jorge et al., 2016) to capture and quantify a wide range of primary and secondary metabolites. Specifically, GC-MS and LC-MS has become fundamental tools to study the biochemical behaviour of plants exposed to pathogen attack (Arbona and Gomez-Cadenas, 2016) due to their unparalleled sensitivity in quantifying many types of phytochemicals.

In plant pathology, metabolomics deals with the profiling of host plant metabolites in response to pathogen infection which helps in the understanding of the host–pathogen interactions through activation/deactivation of metabolites and related signalling pathways (Castro-Moretti et al., 2020). Upon pathogen attack, plants develop different strategies by modifying gene expression and activating metabolic pathways which may accumulate toxic metabolites, thereby killing the pathogen or limiting the damages. Metabolomics has been used to study plant–biotic stress interactions (Tenenboim and Brotman, 2016). The identification of a wide spectrum of compounds synthesized by the plants in response to biotic stresses provides a better understanding of the regulatory processes underlying stress conditions (Badjakov et al., 2012). Over the years, several authors have evaluated the effects of secondary metabolites, glucosinolates, and its hydrolysis products (GHP), conferring resistance to black rot in *Brassica* crops (Table 5). In *B. oleracea*, LC/MS-based metabolite profiling during *Xcc*1 infection revealed the dynamic metabolic changes occurring in the host cell after 48 h of infection, indicating a complex temporal response (Tortosa et al., 2018b). Furthermore, photosynthesis, alkaloids, coumarins, and sphingolipids were shown to play key roles in the metabolic pathways involved in the infection process. Metabolite profiling of a vast number of compounds present in plants could be accomplished by both targeted (Griffiths et al., 2010) and untargeted metabolomic (Schrimpe-Rutledge et al., 2016) approaches. The untargeted approach deals with both the secondary (polyphenols and carotenoids) and primary metabolites, whereas the targeted approach mostly focused on the identification glucosinolates.

**TABLE 5:** Published metabolomic analyses in *Brassica* species involved in resistance against black rot.

Species	Plant organ	Time point (Tissue collection)	Methodology	Objective	Inference	References
Different Brassicaceae crops	Leaves and stems	10-days post-inoculation	Spectrophotometer	To evaluate the potential role of glucosinolates and their hydrolysis products against *Xcc* infection in different crops of Brassicaceae	Various Brassicaceae seedlings shown a correlation between GLS profiles, and their subsequent hydrolysis products, and the inhibition of *Xcc* growth. Positive correlations were found between specific and total GSL contents and the severity of disease.	Aires et al., 2011
*Brassica rapa*	Leaves	0-, 24-, 48-, 72-, 168-h after the inoculation	HPLC	To evaluate the *in vivo* and *in vitro* antibacterial effect of glucosinoates and its hydrolysis products and phenolic compounds against *Xcc*	Gluconapin plays a role in the constitutive resistance to *Xcc*. Gluconapin, some flavonoids and hydroxycinnamic acids were induced by *Xcc* infection.	Velasco et al., 2013
*Brassica napus*	Leaves	14-days post- inoculation	HPLC–ESI–MS/MS	To elucidate the cultivar variation in disease susceptibility and disease responses in relation to hormonal status in the interactions of *Brassica napus* cultivars and *Xcc*	Cultivar variation in susceptibility to infection by *Xcc* is determined by the enhanced alteration of the SA/JA ratio with the antagonistic suppression of JA-regulated gene, as a negative regulator of redox status and phenylpropanoid synthesis in the *Brasica napus*–*Xcc* pathosystem	Islam M.T. et al., 2017
*Brassica oleracea* var. *capitata*	Leaves	0-, 1-, 3-, 5-, 7-, 9-, and 11-days after inoculation (DAI)	Spectrophotometer	To gain a better understanding of the interaction between *Xcc* and cabbage and the changes in the expression of the key defence-related enzymes in compatible interaction	The susceptibility of cabbage to *Xcc* is correlated to the declination of phenylalanine ammonia lyase (PAL) and phenolic contents	Barman et al., 2015
*B. oleracea* var. *italica*	Leaves	3- and 12-days post-infection	UHPLC-QTOF	Metabolite profiling of *B. oleracea* challenged with *Xcc*	The number of compounds belonging to coumarin family changed during *Xcc* infection, especially during the early response. Alkaloid metabolism was modified in response to infection in both 3-and 12-dpi.	Tortosa et al., 2018a
*Brassica oleracea*	Leaves	1-, 2-, 3-, 6-, and 12-days post-inoculation	LC/MS	To unravel the metabolomic response of *B. oleracea* infected with *Xcc* race 1	*Xcc* infection caused dynamic changes in the metabolome of *B. oleracea*. Alkaloids, coumarins or sphingolipids, were postulated as promising key candidates in the infection response	Tortosa et al., 2018b
*Brassica rapa* var. *pekinensis*	Leaves	12-days post-inoculation (DPI)	RP-HPLC	To characterize p-coumaric acid (pCA) induced soluble and cell wall-bound phenolic metabolites in relation to resistance against *Xcc*	Lower disease symptom development in *p*CA-pretreated leaves was associated with a higher accumulation of hydroxycinnamic acids and flavonoids, and vice-versa in MDCA- and non-pretreated (control) leaves.	Islam et al., 2018
*Brassica napus*	Leaves	7-days post- inoculation	HPLC–ESI–MS/MS	Role of p-Coumaric acid against resistance to black rot disease in *Brassica napus*	Treatment with *p*CA primed the JA signalling-mediated induction of phenylpropanoid biosynthesis to provoke disease resistance in *B. napus* against *Xcc*	Islam et al., 2019a
*Brassica napus*	Leaves	14-days post- inoculation	HPLC–ESI– MS/MS, RP-HPLC	To investigate the hormonal regulations in soluble and cell wall-bound phenolic compound accumulation in the resistant and susceptible cultivar of *Brassica napus*	Enhanced JA levels and signalling in resistant cultivar was associated with a higher accumulation of hydroxycinnamic acids and flavonoids, particularly in the cell wall-bound form, and vice versa in the susceptible cultivar with enhanced SA-, ABA-, and CK- levels and signalling.	Islam et al., 2019b
*Brassica oleracea* var. *acephala*	Leaves	21-days post- inoculation	UHPLC	To explore the role of major glucosinolates such as SIN, GIB, GBS in the defense of Kale against *Xcc*	Increasing the amount of GSLs didn’t always result in resistance. Indolic GSL (glucobrassicin) was found inhibitory to infection by *Xcc*	Madloo et al., 2019
*Brassica oleracea* var. *capitata*	Leaves	1-, 3-, and 5-days after inoculation (DAI)	HPLC	To understand the role of glucosinolate biosynthesis and breakdown-related genes for resistance against *Xcc* in cabbage	Four aliphatic (glucoiberverin, sinigrin, gluconapin, and glucoerucin) and four indolic (glucobrassicin, methoxyglucobrassicin, hydroxyglucobrassicin, and neoglucobrassicin) glucosinolates were found positively associated with black rot resistance in cabbage	Rubel et al., 2020
*Brassica napus*	Leaves	14-days post- inoculation (DPI)	HPLC-ESI-MS/MS	To decipher R-gene-mediated calcium signalling and hormonal signalling involved in Effector-triggered immunity (ETI) or susceptibility in the *Xcc–B. napus* pathosystem	JA induction with an antagonistic depression of SA would be part of the resistance mechanism against *Xcc*	Mamun et al., 2020

Glucosinolates (GSLs) are the unique components of metabolome (Verkerk et al., 2009) found in *Brassica* vegetables which are structurally highly diverse and mainly fall into three classes such as aliphatic, indole, and aromatic glucosinolates. GSLs are involved in defence mechanisms against plant pathogens, insects, and nematodes (Bennett and Wallsgrove, 1994; O’Callaghan et al., 2000; Buskov et al., 2002; Serra et al., 2002; Kliebenstein, 2004). *Brassicaceae* family is mostly dominated by aliphatic and indolic glucosinolates (Fahey et al., 2001; Bekaert et al., 2012). The breakdown products of both aliphatic and indolic glucosinolates by an endogenous myrosinases enzyme (*b*-thioglucoside glucohydrolases) were found to have anti-fungal and anti-bacterial properties in different crops (Manici et al., 1997; Agerbirk et al., 1998; Brader et al., 2001; Tierens et al., 2001; Stotz et al., 2011; Calmes et al., 2015). Several *in vivo* and *in vitro* experiments were conducted to estimate the effect of GSLs and their hydrolysed products (GHP) for disease resistance in *Brassica* crops. Transgenic *A. thaliana* with modified glucosinolates profile enhanced the resistance against *Erwinia carotovora* and *Pseudomonas syringae* pv. *maculicola* (Brader et al., 2006). In *B. napus*, cultivars with higher GSL content showed resistance against *Alternaria* spp. and *Leptosphaeria maculans* compared to cultivars with low GSLs (Giamoustaris and Mithen, 1995). The *in vitro* effect of GHP, especially different isothiocyanates (benzylisothiocyanate, 2-phenylethylisothiocyanate, the isothiocyanate mix and sulforaphane) were found effective against six plant pathogenic bacteria such as *Agrobacterium tumefaciens*, *Erwinia chrysanthemi*, *Pseudomonas cichorii*, *Pseudomonas tomato*, *Xanthomonas campestris*, and *Xanthomonas juglandin* (Aires et al., 2009). Though several research projects were dedicated to elucidate the role of GSLs and GHP against fungal pathogens, fewer studies were conducted with pathogenic bacteria and much less with *Xcc* (Table 5). Several studies have stated that indolic GSLs are related to the resistance to necrotrophs, biotrophs, and hemibiotrophs in *Brassica* crops (Hiruma et al., 2013). *Xcc* being a hemibiotropic pathogen, the role of GHP was evaluated against *Xcc* infection in various *Brassicaceae* seedlings and positive correlations were found between specific and total GSL contents and the severity of *Xcc* infection, though no significant correlations were reported between *Xcc* infection and total phenolics (Aires et al., 2011). Nevertheless, the susceptibility of the *Brassica* plants against *Xcc* was found to be higher in plants with lower contents of aromatic-GSLs and glucoraphanin, both acting as inhibitors of *Xcc*. This necessitates further requirements of clear and detailed *in vitro* studies to evaluate the role of GSLs and its GHP in defence mechanisms against *Xcc*. So, experiments were carried out to evaluate the *in vivo* and *in vitro* antibacterial activities of gluconapin, its isothiocyanate (ITC) against *Xcc* type 4 in *B. rapa* (Velasco et al., 2013). The results demonstrated gluconapin and its ITC varieties possessing antibacterial effect on the development of Xanthomonas, and gluconapin playing a role in imparting constitutive resistance to *Xcc*. Additionally, the methanolic extracts of *B. rapa* containing glucosinolates and phenolic compounds curbed the growth of *Xcc*. Though GSLs and its hydrolytic products show a wide range of antimicrobial activities, our knowledge on the role of specific metabolites in defending the *Brassica* crops and their interactions with the pathogens is poorly understood. So, the individual role of two aliphatic (sinigrin, glucoiberin) and one indolic (glucobrassicin) GSLs against *Xcc* was explored in *B. oleracea* var. *acephala* L. (kale) and the indolic GSL glucobrassicin was found inhibitory to *Xcc* infection than the aliphatic GSLs (Madloo et al., 2019). Interestingly, the results indicated that increasing the amount of a particular GSL may not always result in disease resistance. Instead, its effects are dependent on the pathogen and the type of GSLs. Also, factors like modification of the metabolites during pathogen infection may regulate its affect in inhibiting the pathogens. This is noteworthy as the host genotypes exhibit different GSL profiles and concentrations in different genetic backgrounds.

The variation in GSL contents could happen due to the allelic variation of GSL biosynthesis genes (Rangkadilok et al., 2002). In *B. oleracea*, three loci (GSL-PRO, GSL-ELONG, and GSL-ALK) were reported to regulate the aliphatic GSL profile (Li et al., 2001). In *Arabidopsis*, upon fungal infection, the *CYP81F2* gene is expressed resulting in the hydrolysis of GSLs by PENETRATION2 (PEN2) and accumulated as indolic 4-methoxy-glucobrassicin in cells (Bednarek et al., 2009). But the information about GSL-regulated resistance mechanisms, expression profiling of GSL biosynthesis genes, and interaction between GSL profiles of black rot susceptible and resistant plants is scanty in *B. oleracea*. In a recent study, the relative expression of 43 GSL biosynthesis and breakdown-related genes were estimated upon *Xcc*4 inoculation in cabbage (Rubel et al., 2020). In the resistant lines, nine genes showed consistent expression patterns. Expression values of two (*ST5c-Bol030757* and *AOP2-Bo9g006240*) and five genes (*MYB34-Bol017062*, *MYB122-Bol026204*, *CYP81F2-Bol012237*, *CYP81F4-Bol032712*, and *CYP81F4-Bol032714*) showed positive association with aliphatic and indolic GSL compounds, respectively. Finally, four aliphatic (glucoiberverin, sinigrin, gluconapin, and glucoerucin) and four indolic (glucobrassicin, hydroxyglucobrassicin, methoxyglucobrassicin, and neoglucobrassicin) compounds were found positively associated with black rot resistance which may help in elucidating the role of GSL biosynthesis and breakdown-related genes in conferring resistance to *Xcc* in cabbage.

The phytohormones, such as JA and SA, are important central defence signalling molecules which maintain complex interactions with each other and other hormones such as ABA, cytokinin, gibberellic acid, and indole acetic acid to regulate defence responses (Anderson et al., 2004; Martínez-Medina et al., 2017). The antagonistic interaction between SA and JA and synergistic interaction of JA with ethylene have been well documented in previous studies (Pieterse et al., 1996; Van Wees et al., 2000). So, elucidation of cultivar variation in relation to hormonal status is significant determinant of disease susceptibility and resistance to *Xcc.* In a study, increased ratios of ABA/JA and SA/JA increased the susceptibility to *Xcc* in the *B. napus* cultivars which acted as a negative regulator of redox status and phenylpropanoid synthesis required for inducing resistance (Islam M.T. et al., 2017). With the enhanced expression of SA signalling regulatory gene *NPR1* and transcriptional factor *TGA1*, the ratios of ABA/JA and SA/JA increased with the antagonistic suppression of JA-regulated gene *PDF 1.2*, leading to higher susceptibility of the cultivars. Similarly, Mamun et al. (2020) also reported the R-gene-mediated induction of JA with an antagonistic depression of SA for ETI response suggesting the importance of maintaining proper SA/JA ratio in conferring resistance against *Xcc* in *B. napus* (Figure 3A).

**FIGURE 4:**
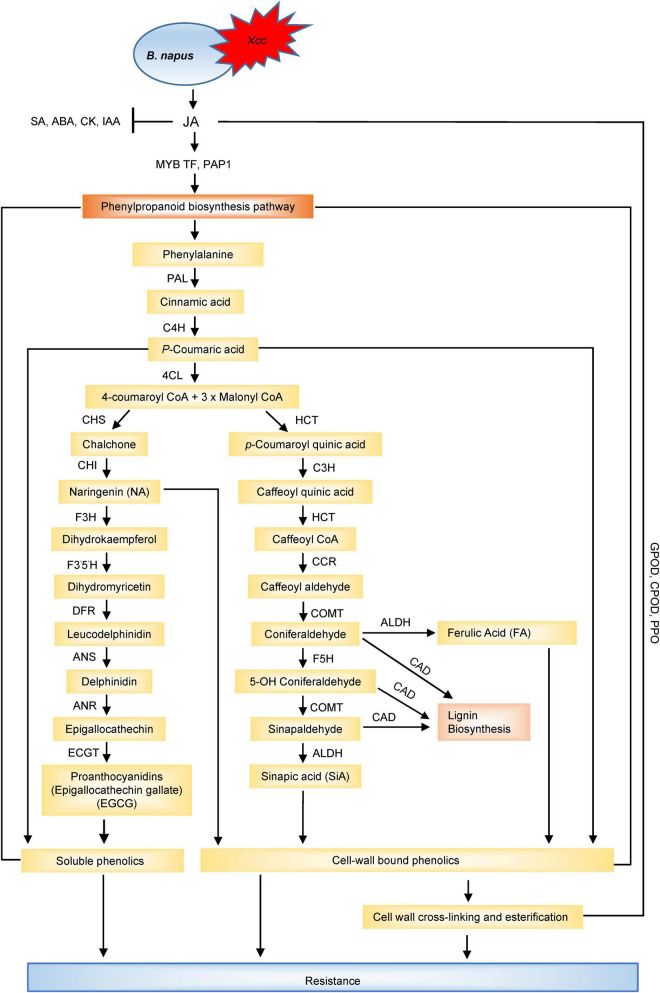
Schematic view of a model depicting Jasmonic acid **(JA)**-mediated phenylpropanoid biosynthesis pathway and hormonal regulations of phenolic accumulation (soluble and cell-wall bound) in relation to resistance against *Xcc* in *Brassica napus* adopted from previous studies (Islam et al., 2018, 2019b). JA, jasmonic acid; SA, salicylic acid; ABA, abscisic acid; CK, cytokinin; IAA, indoleacetic acid; MYB TF, MYB transcriptional factor; PAP1, production of anthocyanin pigment 1; PAL, phenylalanine ammonia-lyase; C4H, cinnamate-4-hydroxylase; 4CL, 4-coumaroyl CoA-ligase; CHS, chalcone synthase; CHI, chalcone isomerase; F3H, flavanone 3- hydroxylase; F3’5’H, flavonoid 3’,5’hydroxylase; DFR, dihydroflavanol 4-reductase; ANS, anthocyanidin synthase; ANR, anthocyanidin-reductase; ECGT, epicatechin:1-*O*-galloyl-β-D-glucose-*O*-galloyltransferase; HCT, hydroxycinnamoyl-CoA:shikimate hydroxycinnamoyl transferase; C3H, coumarate 3-hydroxylase; CCR, cinnamyl CoA reductase; COMT, caffeic acid O methyltransferase; ALDH, aldehyde dehydrogenase; F5H, ferulate 5- hydroxylase; CAD, cinnamyl alcohol dehydrogenase; GPOD, guaiacol peroxidases; CPOD, coniferyl alcohol peroxidase; PPO, polyphenol oxidase.

Also, plant phenolics are involved in disease resistance mechanisms in different pathosystems which are synthesized by the phenylpropanoid pathway (Dixon et al., 2002) through the conversion of *p*-coumaric acid (*p*CA) into hydroxycinnamic acids and flavonoids (Cartea et al., 2011b). Islam M.T. et al. (2017) reported higher accumulation of flavonoids, hydroxycinnamic acids, and proanthocyanidins in the resistant cultivar (cv. Capitol) by the enhanced expression of the phenylpropanoids biosynthesis-related genes (*CHS, F5H*, and *ANR*) upon *Xcc* infection. Furthermore, expression of these genes were found positively correlated with the enhanced expression of JA-signalling gene *PDF 1.2* resulting in the elevated resistance. This clearly demonstrated that JA positively regulates the phenylpropanoid synthesis pathway and JA-signalling pathway is involved in conferring resistance against hemibiotrophic pathogen like *Xcc*. The pre-treatment of Chinese cabbage genotypes with *p*-coumaric acid (regulates the phenylpropanoid biosynthesis pathway) alleviated the *Xcc* symptoms with higher accumulation of hydroxycinnamic acids (ferulic acid and sinapic acid) and flavonoids (EGC and EGCG) both in soluble and cell wall-bound form (Islam et al., 2018). The pre-treatment with *p*-coumaric acid also enhanced the expression of *CHS* and *HCT* genes which regulate the synthesis of flavonoids and hydroxy cinnamic acids, respectively, in the phenylpropanoid biosynthesis pathway. The hormonal regulation of *p*CA-induced resistance against *Xcc* was investigated by Islam et al. (2019a) who revealed the enhancement of JA content and expression of signalling genes (*COI1* and *PDF1.2*) in plants pre-treated with *p*CA in *B. napus* cultivars inoculated with *Xcc*. In the *Xcc*-inoculated plants, a higher accumulation of total hydroxycinnamic acids and proanthocyanidins were reported along with the up-regulation of phenylpropanoids synthesis-related genes in the resistant cultivar. To further investigate if hormonal regulation is associated with the higher accumulation of defensive metabolites synthesized by phenylpropanoid pathway, Islam et al. (2019b) reported that enhanced JA levels and signalling positively regulated the accumulation of phenolic metabolites (*p*CA, SiA, FA, NA, and EGCG) in the cell wall-bound form and was associated with antagonistic depression of SA, ABA, CK, and IAA-signalling genes in *B. napus* upon *Xcc* inoculation (Figure 4). This was accompanied by up-regulation of *PAP1* (production of anthocyanin pigment 1)-induced phenylpropanoid biosynthesis genes.

Induced resistance mechanisms are also associated with lignification (Simard et al., 2001) and esterification of cell wall polysaccharides by phenolics (especially with *pCA*, ferulic acid, and sinapic acid) (Azuma et al., 2005; Cvikrová et al., 2006) under pathogen attack. The enhanced activity of two isozymes of peroxidases, guaiacol peroxidase (GPOD), and coniferyl alcohol peroxidase (CPOD) involved in cell wall cross-linking in the resistant cultivar of *B. napus*, ultimately induced resistance against *Xcc* (Islam et al., 2019b).

## Key Genes and Pathways Related to Black Rot Resistance in *Brassica* spp.

**FIGURE 5:**
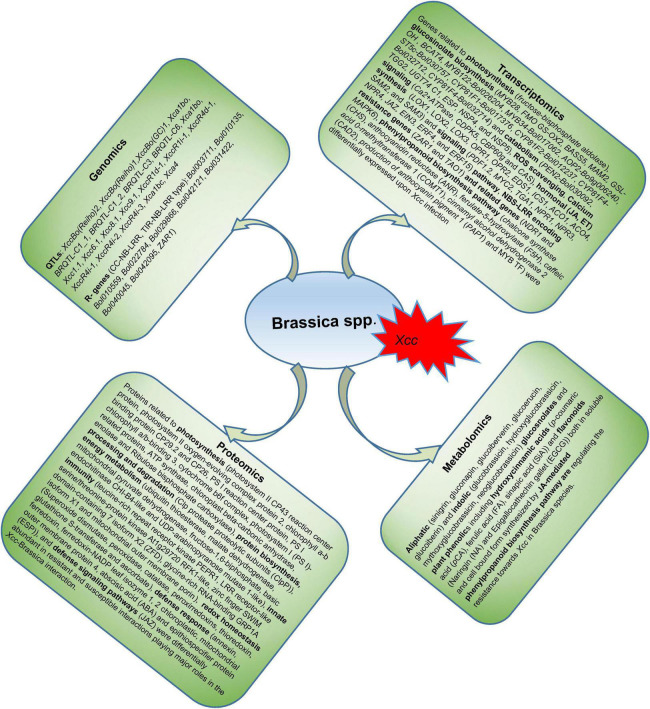
Schematic diagram representing key genes and pathways involved in *Xcc* resistance in *Brassica* species.

*Xcc* needs both living and dead tissues for growth and reproduction and is a hemibiotrophic pathogen behaving both as a biotrophic and necrotrophic pathogen. In response to *Xcc* attack, in *B. oleracea*, the first layer of basal defence is conferred by pathogen-associated molecular pattern (PAMP)-triggered immunity (PTI) which is overcome by *Xcc* by delivering the effector proteins into plant cells leading to the suppression of the basal defence of the host. Then, the second level of defence, called ETI, is mediated by the intracellular receptors of the host encoded by the NBS-LRR type R-genes which bind to the pathogen effector proteins and inhibit the bacteria development. In this review, we have discussed the genomics, transcriptomics, proteomics, and metabolomics basis of key genes, proteins, and metabolites involved in the effective responses against *Xcc* (Figure 5). The genomic studies revealed that the black rot resistance is mostly a complex trait and is controlled by QTLs with minor effects (Table 2) though few scholars have reported major effect QTLs. While secondary metabolites like GSLs were involved in quantitative resistance against *Xcc* in *B. oleracea*, several NBS-LRR type R-genes were identified regulating defence response against *Xcc*. One CC-NB-LRR type R-gene, *ZAR1* regulated ETI response to *Xcc* by co-ordinating SA and JA synthesis in *B. napus* (Figure 3A). Transcriptomic analysis revealed the genes related to photosynthesis (*fructose-bisphosphate aldolase*), glucosinolate biosynthesis (*MYB122-Bol026204*, *MYB34-Bol017062*, *AOP2-Bo9g006240*, *ST5c-Bol030757*, *CYP81F1-Bol017376*, *CYP81F2-Bol012237*, *CYP81F4-Bol032712*, *CYP81F4-Bol032714*, *MYB28*, *FMO GS-OX2*, *BASS5*, *MAM2*, *GSL-OH*, and *BCAT4*) and catabolism (*PEN2-Bol030092*, *TGG2*, *UGT74 C1*, *ESP*, *NSP4*, and *NSP5*), phenylpropanoid biosynthesis pathway [*chalcone synthase* (*CHS*), *anthocyanidin reductase* (*ANR*), *ferulate-5-hydroxylase* (*F5H*), *caffeic acid 0-methyltransferase 1* (*COMT1*), *cinnamyl alcohol dehydrogenase 2* (*CAD2*), *production of anthocyanin pigment 1* (*PAP1*) and MYB transcription factor], ROS scavenging, calcium signalling-related genes (*Ca2+ATPase, CDPK5, CBP60g* and *CAS*), hormonal synthesis (*LOX1*, *LOX2*, *LOX3*, *OPR1*, *OPR2*, *EDS1, ICS1*, *ACO1*, *ACO4*, *SAM2*, and *SAM3*) and signalling pathway (*PDF1.2*, *MYC2*, *TGA1*, *NPR1*, *NPR3*, *NPR4*, *JAZ*, *EIN3*, *ERF4*, and *ERF15*), resistance (*ZAR1*, *TAO1*), and related genes (*NDR1*, *MAPK6*) were differentially expressed upon *Xcc* infection. Proteomic studies identified the proteins related to photosynthesis (photosystem II CP43 reaction centre protein, photosystem II oxygen-evolving complex protein 2, chlorophyll a-b binding protein CP29.2 and CP26, PS I reaction centre protein, PS I chlorophyll a/b-binding 3, cytochrome b6f complex, ATP synthase, chloroplast beta-carbonic anhydrase, enolase and Ribulose bisphosphate carboxylase), protein biosynthesis, processing and degradation [Clp protease proteolytic subunits (ClpP)], energy metabolism (ubiquitin thioesterase, malate dehydrogenase, mitochondrial pyruvate dehydrogenase, fructose-1,6-biphosphate, basic endochitinase CHI-B4-like and UDP-arabinopyranose mutase 1-like), innate immunity [leucine-rich repeat receptor kinase PEPR1, LRR receptor-like serine/threonine-protein kinase At1g29720 RFK1-like and zinc finger SWIM domain-containing 7 isoform X2 (ZFD), glycine-rich RNA-binding GRP1A isoform X1 and mitochondrial outer membrane porin], redox homeostasis (superoxide dismutase, peroxidase, catalase, peroxirredoxins, thioredoxin, glutathione S-transferase and ascorbate), defence response [annexin, ferredoxin, ferredoxin-NADP leaf isozyme 1, 2 chloroplastic, mitochondrial outer membrane protein 4, ABA and epithiospecifier protein (ESP)], and signalling (JAZ) pathways playing major roles in the *Xcc-Brassica* interaction. Specifically, regulation of the photosynthesis-related proteins was involved in both early and later stages of infection by *Xcc*. The decreased abundance of proteins in the resistant plants during early stage of infection suggested that susceptible plants of *Brassica* spp. require more energy to cope with the infection by *Xcc* in the initial stage. Metabolomic studies indicated that glucosinolates and its GHP and plant secondary metabolites, including phenolics synthesized by phenylpropanoid biosynthesis pathway are involved in disease resistance mechanisms against *Xcc* in different *Brassica* species. A number of *in vivo* and *in vitro* studies evaluated the effects of GSLs and their potential role against *Xcc* infection and it could be concluded that aliphatic (sinigrin, gluconapin, glucoiberverin glucoerucin, and glucoiberin) and indolic (glucobrassicin, hydroxyglucobrassicin, mythoxyglucobrassicin and neoglucobrassicin) GSLs present in different tissues of *Brassica* species are associated positively in conferring resistance against *Xcc*. Fine tuning of hormonal signalling plays a significant role to activate immune response against pathogens. Calcium signalling regulate the PTI or ETI in plants (Bigeard et al., 2015) and different calcium signalling genes play potential roles during host defence against *Xcc* (Tortosa et al., 2019). R-genes played significant role to induce different hormonal signalling through calcium signalling during *Brassica*–*Xcc* interaction (Mamun et al., 2020). The phytohormones, SA and JA, are regarded as the central regulators in host defence against biotrophic and hemibiotrophic pathogens, respectively, and are mutually antagonistic (Tortosa et al., 2019; Vega-Álvarez et al., 2021). Several studies conducted by different authors indicated that disease resistance and susceptibility to *Xcc* infection is dependent on alteration of the SA/JA ratio (Islam M.T. et al., 2017; Islam et al., 2019b; Mamun et al., 2020). The enhanced alteration of the SA/JA ratio determined the susceptibility whereas the enhanced JA levels and signalling with antagonistic depression of SA induced resistance against *Xcc* in *Brassica* species. Taken together, we could suggest that JA signalling pathway is playing a critical role in conferring host resistance against *Xcc* in *Brassica* species (Figures 3A,B, 4). Furthermore, the “hormonal crosstalk” among various hormone signalling pathways is believed to be widely involved in *Xcc* resistance in *Brassica* species. In this review, we have observed that JA is mediating the phenylpropanoid biosynthesis pathway to impart resistance toward *Xcc* through the accumulation of defensive metabolites. Increased ratios of SA/JA and ABA/JA up-regulated the SA signalling regulatory genes, thereby negatively regulating phenylpropanoid synthesis. Metabolomic studies have thrown insight on how phenylpropanoid biosynthesis pathway is serving as a rich source of phenolics including hydroxycinnamic acids (*p*-coumaric acid, ferulic acid, and sinapic acid) and flavonoids (naringin and epigallocathechin gallet), both in soluble and cell-wall bound form to confer resistance against *Xcc*.

## Challenges and Future Perspectives

In recent years, most of the plant researchers have focused on host–pathogen interactions as pathogen attack involves many dynamic changes in the host plant which ultimately define the resistance or susceptibility of the plant. Host–pathogen interaction is a complex phenomenon and had always fascinated the plant researchers. Hence, a great research emphasis has been put to understand the mechanisms underlaying resistance. In the recent decades, significant advances were made in understanding the resistance mechanisms for biotic stresses at the molecular level in *Brassica* crops. The complex genetic and molecular processes involved in defence response have necessitated the use of advance technologies to integrate all the biological informations, and to analyse it jointly. The ever-growing demand for improved crop varieties have forced the researchers to adopt omics-based technologies to enhance crop productivity. Integrated multi-omics approaches could provide important insights to understand the complex host–pathogen interactions. Multi-omics approaches are beneficial as it could provide large scale insights into complex plant systems (Suravajhala et al., 2016) instead of using single ‘omic’ alone.

Often, protein and metabolite expression differ with that of mRNA expression of the corresponding genes. Therefore, proteomic and metabolomic research should be given due importance as they are the end products of the central dogma. If these approaches are integrated correctly at the right scale, they may reveal a multi-dimensional view of the plant diseases (Crandall et al., 2020). Multi-omics technologies combined with the computational systems may identify the mimicking molecules in the host and/or pathogen triggering the plant defence systems (Pathak et al., 2017). In recent years, breakthroughs in omics technologies have been made in several model plants, but such applications are still in the infancy stage in *B. oleracea*. Most of the ‘omics’ research in *Brassica* species have focused on fungal diseases than the bacterial or viral pathogens. The availability of draft genome sequence and the advancement of sequencing technologies have triggered substantial progress in genomics and transcriptomics in *B. oleracea*, but the two other major branches of ‘omics,’ proteomics and metabolomics, are lagging behind. So, the questions and bottlenecks which restrict the implementation of omics approaches for black rot resistance need to be studied.

The inheritance of black rot resistance is complex and contradictory which act as a bottleneck in a conventional breeding programme. Compared to *B. oleracea*, related *Brassica* species such as ‘A’ and ‘B’ genomes are the potential sources of black rot resistance. Furthermore, the favourable alleles of diverse resistance genes present in the wild relatives of *B. oleracea* need to be screened to explore how the resistance gene expression is carried out by regulators such as small RNAs (Zhang et al., 2019). The process of utilizing wild diversity need to be fast-tracked so that genomic part of the loci linked to QTLs for black rot resistance could be explored. The identification of new resistance genes in alien *Brassica* species and availability of linked markers will assist in the development of pre-breeding black rot resistant genetic stocks/lines through marker-assisted backcross breeding in *B. oleracea*.

Due to large-scale whole-genome resequencing, sequence-based markers such as SNPs and InDels have been generated which could be the basis of large application of GWAS and bi-parental QTL mapping projects for disease resistance, especially for black rot in *B. oleracea*. Additionally, the validated markers need to be converted to ‘breeder-friendly’ markers for effective use in marker-assisted selection programme. Several methods like eQTL/eGWAS, pQTL/pGWAS, and mQTL/mGWAS where transcript, protein, and metabolite profiles serve as phenotypic information to detect the loci controlling their expression levels can be used to understand the *Xcc-B. oleracea* pathosystem. These approaches have been successfully utilized in several species for crop improvements (Zhu et al., 2016; Fang and Luo, 2019; Gu et al., 2019). Although several QTL mappings were conducted and a number of genes for black rot resistance were reported in different *Brassica* species, none has been cloned. So, future efforts in the direction of map-based cloning are essential to identify the candidate genes responsible for black rot resistance. Furthermore, other mapping approaches such as MutMap (Abe et al., 2012), MutMap+ (Fekih et al., 2013), and MutMap-Gap (Takagi et al., 2013) could be utilized effectively to identify the loci responsible for black rot resistance. Also, due to prevalence of many races in black rot, gene pyramiding strategy will aid in the development of *B. oleracea* with durable resistance.

Development of high-throughput phenotyping platforms, establishment of robust glasshouse-based phenotyping methods, and phenotyping of large populations across environments and developmental stages during disease progress are the bottlenecks in resistance breeding. So, introduction of phenomics via advanced sensors, cameras, robotics, and image analysis tools holds promise in revealing the genetic mechanisms underlying disease resistance (Houle et al., 2010). Also, targeting the whole crop system via quantitative and automated screening and selection methods could be possible through the advancement of omics technology bridging the gap between the genotype to phenotype. Omics technologies have the potential to develop ‘smart crops’ being sustainable for abiotic and biotic stresses with higher productivity.

Gene editing technologies *viz.* CRISPR/Cas, zinc finger nucleases (ZFNs), and transcription activator like effector nucleases (TALENs) are promising tools which have enormous potential to boost resistance against black rot. Using CRISPR/Cas9 system, functional analysis of candidate genes regulating resistance against *S. sclerotiorum* was carried out in *B. napus* (Sun et al., 2018). The knockout of the *BnWRKY70* gene encoding WRKY transcription factors in *B. napus* exhibited enhanced resistance to sclerotinia. Genomic selection may prove to be a powerful approach for molecular breeding of disease resistance governed by minor genes (Poland and Rutkoski, 2016). So, the scope of implementing genomic selection for black resistance to develop high quality resistant varieties need to be explored in *B. oleracea*, which is basically a quantitative trait. A deep insight into epigenetics could reveal how it affects the response of a plant to pathogen attack, thereby adding a new dimension to understand the host–pathogen interactions. Transposable elements were reported to contribute to disease resistance and susceptibility in *Brassica* crops (Alonso et al., 2019; Tirnaz and Batley, 2019). In *Arabidopsis*–*P. brassicae* pathosystem based epigenotyped epigenetic RILs, DNA methylation was found to be contributing towards quantitative resistance against clubroot (Liégard et al., 2019). Exploring the epigenetic variability regulating the phenotypic response of *B. oleracea* towards black rot would be exciting.

Insufficient knowledge of the omics tools, especially proteomics and metabolomics, may act as a significant barrier for functional characterisation of black rot resistance genes. Metabolomics provide a broader, deeper, and an integral perspective of metabolic profiles in stressed conditions. In *B. oleracea*, metabolomic research has mostly focused on glucosinolates. Therefore, the precise identification of leftover metabolites remains a challenge. Also, availability of low number of metabolites is another bottleneck in the identification of metabolites involved in highly complex mechanisms like host–pathogen interactions. Mathematical models can support the metabolic pathway analysis to study the behaviour of cells carrying out a particular function in multicellular organisms (Neik et al., 2020). Through system biology, a genome-scale metabolic model of *S. sclerotiorum* was made to assess the metabolic activity in several components of the hyphal cells of *A. thaliana*–*S. sclerotiorum* pathosystem which revealed that cooperation in *S. sclerotiorum* hyphal cells is essential for host colonisation and virulence (Peyraud et al., 2019). The metabolic pathway for JA signalling in the *Brassica*–*Alternaria* pathophysiology was modelled through system biology to identify important elements regulating the resistance mechanisms in *Brassica* crops (Pathak et al., 2017).

The omics era has taught us to follow a holistic approach while interpreting the complex biological systems starting from molecular to cellular level (Pathak et al., 2018). As the cost of the omics analyses continues to decrease, different high-throughput omics data are made available. But this has posed a complex challenge to the scientific community as we face difficulties in integrating different kinds of extensive omics data sets which come in different formats requiring the expertise to pre-process, analyse, and interpret the final results (Crandall et al., 2020). These challenges including storage, reproducibility, lack of reference database, and reference phytochemicals for metabolomics can be addressed through collaborative efforts, so that the potential of multi-omics research can be realized. We need improved statistical models with modern computing power to scale up the capabilities and efficiency in integrating the full omics datasets. No single research group can handle the multi-omics data generation on their own. Multi-institutional and cross-team research collaboration can allow information exchange, communication between researchers, integration of expertise and may create breakthroughs and answer the unsolved questions. In the era of genomic revolution, with the emergence of multi-omics platforms, we are moving toward a new era of ‘big biological data.’ So, development of integrative omics data management systems, bioinformatics tools and algorithms, and building of accessible databases is the need of the hour to address the ever-growing need for big data analysis (Su et al., 2019). Also, publicly available data should be used sensibly necessitating the standardisation of commonly used omics technologies (Hasin et al., 2017). For instance, publishing of new genome version, transcript annotation on a regular basis makes it challenging to compare the RNA-seq expression studies. So, the successful deployment of omics technologies not only require technological advances and minimal analytical challenges, but also need a conceptual shift in the research paradigm (Hasin et al., 2017). While continuous efforts are going on to coordinate between disciplines such as genomics, transcriptomics, proteomics and metabolomics of the plant systems, a new discipline ‘crop system biology’ has been proposed (Yin and Struik, 2007). Through crop system biology, the physiological and metabolic complexity of the plant diseases can be studied to understand complex interactions between host and pathogen which may unveil the pathways and regulatory networks involved. It could be a complementary approach to connect functional genomics and crop modelling to assist plant breeding in improving the disease resistance of major crops.

With the flooding of enormous omics data, the integration of omics data will lead to the identification of many genes simultaneously for disease resistance thereby shifting the research paradigm from single gene analysis to pathway or network analysis. This may revolutionize the future crop breeding system and ensure the development of disease resistant crops for sustainable agriculture. Furthermore, computational approaches could be implemented to construct network biology utilizing the great amount of data collected through multi-omics technologies to understand the plant-pathogen interactions (Botero et al., 2018). No doubt, this will be highly computer-intensive, but it will be handy as a powerful tool for *in silico* assessment of crop response under various pathogen attacks. The phenomenal progress in genomics should prompt the plant researchers to adopt all the innovative approaches to address the important questions in developing disease resistant cultivars for sustainable agricultural production.

## Conclusion

Omics tools have demonstrated tremendous potential in advancing our knowledge on the host-pathogen interactions and has opened up new possibilities to rapidly identify QTLs/candidate genes, pathogenicity-related genes, metabolic pathways, and proteins, prompting in-depth cellular and biochemical understanding of resistance mechanisms against a range of pathogens. Through an integrated multi-omics approach, numerous datasets at the genomics, transcriptomics, proteomics, and metabolomics level have been used to elucidate the complex mechanisms and pathways regulating black rot resistance in *B. oleracea.* Genomic studies revealed that the black rot resistance is mostly a complex trait and is governed by QTLs with minor effects except few major effect QTLs. Transcriptomic analysis divulged the genes related to photosynthesis, glucosinolate biosynthesis and catabolism, phenylpropanoid synthesis, ROS scavenging, calcium signalling, hormonal synthesis, and signalling pathway genes being differentially expressed upon *Xcc* infection. Due to the availability of sequencing data, substantial progress has been made both in genomics and transcriptomics, but the knowledge on proteins and metabolites directly involved in the complex mechanisms of resistance against black rot is minimal. But the identification of proteome components is a fast-moving area which, by the help of modern analytical techniques like MS platforms, are going to be used widely in *Brassica* species to provide more information on post-transcriptional modifications. Comparative proteomic analysis identified the involvement of proteins related to photosynthesis, protein biosynthesis, processing and degradation, energy metabolism, innate immunity, redox status, and defence response and signalling pathways in *Xcc*–*Brassica* interaction. Specifically, most of the studies focused on the regulation of the photosynthesis-related proteins as a resistance response in both early and later stages of infection. Also, information about the composition of metabolites could be a powerful mean for improvement of *B. oleracea* for disease resistance. Metabolomic studies suggested that glucosinolates (aliphatic and indolic) and its GHP and phenolics such as hydroxycinnamic acids and flavonoids synthesized by the JA-mediated phenylpropanoid pathway are involved in disease resistance mechanisms against *Xcc* in *Brassica* species. Phytohormone studies indicated that JA signalling pathway is regulating resistance against hemibiotrophic pathogen like *Xcc* in *Brassica* species. Using of multi-omics tools will definitely speed up the screening of superior alleles responsible for resistance against major pathogens including black rot in *B. oleracea* and may unveil new horizons for future research. Integration of data from different hierarchies using different mathematical and statistical models will lead to new discoveries involved in crop improvements and will provide ease to the plant breeders. The bonhomie between multi-omics technologies and plant breeding is going to trigger major breakthroughs in the crop improvement and can have the maximum benefits from the minimum in the years to come.

## Author Contributions

HG conceived the idea and revised the manuscript. RS wrote the manuscript. HG, ZZ, YS, XS, JW, and HY reviewed and edited the manuscript. All the authors have discussed and agreed to the published version of the manuscript.

## Conflict of Interest

The authors declare that the research was conducted in the absence of any commercial or financial relationships that could be construed as a potential conflict of interest.

## Publisher’s Note

All claims expressed in this article are solely those of the authors and do not necessarily represent those of their affiliated organizations, or those of the publisher, the editors and the reviewers. Any product that may be evaluated in this article, or claim that may be made by its manufacturer, is not guaranteed or endorsed by the publisher.

## Footnotes

1
http://brassicadb.cn
